# Love-hate relationship between hepatitis B virus and type 2 diabetes: a Mendelian randomization study

**DOI:** 10.3389/fmicb.2024.1378311

**Published:** 2024-04-05

**Authors:** Yunfeng Yu, Keke Tong, Gang Hu, Xinyu Yang, Jingyi Wu, Siyang Bai, Rong Yu

**Affiliations:** ^1^The First Hospital of Hunan University of Chinese Medicine, Changsha, China; ^2^The Hospital of Hunan University of Traditional Chinese Medicine, Changde, China; ^3^College of Chinese Medicine, Hunan University of Chinese Medicine, Changsha, China; ^4^The Third School of Clinical Medicine, Zhejiang Chinese Medical University, Hangzhou, China

**Keywords:** hepatitis B virus, chronic hepatitis B, liver fibrosis, liver cirrhosis, type 2 diabetes, Mendelian randomization

## Abstract

**Objective:**

The impact of hepatitis B virus (HBV) on the risk of type 2 diabetes (T2D) remains a controversial topic. This study aims to analyze the causal relationship between HBV and T2D using Mendelian randomization (MR).

**Methods:**

Single nucleotide polymorphisms on chronic hepatitis B (CHB), liver fibrosis, liver cirrhosis, and T2D were obtained from BioBank Japan Project, European Bioinformatics Institute, and FinnGen. Mendelian randomization was utilized to evaluate exposure-outcome causality. Inverse variance weighted was used as the primary method for MR analysis. To assess horizontal pleiotropy and heterogeneity, we conducted MR-Egger intercept analysis and Cochran’s *Q* test, and the robustness of the MR analysis results was evaluated through leave-one-out sensitivity analysis.

**Results:**

MR analysis revealed that CHB was associated with a decreased genetic susceptibility to T2D (OR, 0.975; 95% CI, 0.962–0.989; *p* < 0.001) while liver cirrhosis (OR, 1.021; 95% CI, 1.007–1.036; *p* = 0.004) as well as liver cirrhosis and liver fibrosis (OR, 1.015; 95% CI, 1.002–1.028; *p* = 0.020) were associated with an increased genetic susceptibility to T2D. MR-Egger intercept showed no horizontal pleiotropy (*p* > 0.05). Cochran’s *Q* showed no heterogeneity (*p* > 0.05). Leave-one-out sensitivity analysis showed that the results were robust.

**Conclusion:**

CHB has the potential to act as a protective factor for T2D, but its effectiveness is constrained by viral load and disease stage. This protective effect diminishes or disappears as viral load decreases, and it transforms into a risk factor with the progression to liver fibrosis and cirrhosis.

## 1 Introduction

Type 2 diabetes (T2D) is a metabolic disease characterized by insulin resistance and abnormally elevated blood glucose (Prabhakar and Bhuvaneswari, 2020). Epidemiological research have shown that T2D affected 6,059 per 100,000 people globally in 2017, and the prevalence of T2D is projected to increase to 7,079 per 100,000 people by 2030 (Khan et al., 2020). T2D is primarily characterized by persistent hyperglycemic, which is a major factor of serious complications in organs such as the cardiovascular system and kidneys (Stewart, 2022). It has been reported that more than one million deaths per year are related to diabetes and it has become the ninth leading cause of death worldwide (Khan et al., 2020). Common risk factors for T2D mainly include poor diet, physical inactivity, smoking, alcohol consumption, and obesity (Ismail et al., 2021). Focusing on related risk factors, especially some chronic diseases, is relevant for the prevention and treatment of T2D.

Chronic hepatitis B (CHB) is a chronic infectious disease caused by infection with the hepatitis B virus (HBV) (Li et al., 2022). Epidemiological research have shown that approximately 260 million people worldwide are chronically infected with HBV (Paccoud et al., 2019). Chronic HBV infection is a major cause of liver fibrosis, cirrhosis and hepatocellular carcinoma, and has become a serious global public health problem (Lee and Ahn, 2016; Ginès et al., 2022). The therapeutic goal of CHB is to achieve sustained suppression of HBV DNA to prevent progression to liver cirrhosis and hepatocellular carcinoma (Ayoub and Keeffe, 2011). Previous studies have shown that HBV infection is associated with a 33% increased risk of diabetes and is a potential diabetes-related risk factor (Cai et al., 2015). However, other researchers have found that the increased risk of T2D is caused by cirrhosis of hepatitis B rather than HBV infection (Zhang et al., 2015), and there have even been suggestions that HBV infection may be a potential protective factor for T2D (Li et al., 2016). The association between HBV infection and T2D is a controversial topic, and they present an intricate love-hate relationship. Elucidating the potential role of HBV on T2D will help clinicians to better formulate strategies for the prevention and treatment of T2D.

Mendelian randomization (MR) is an emerging method of epidemiological research that assesses the causal relationship between exposure and outcome through genetic variation, effectively avoiding confounding factors (Sanderson, 2021). This study employed MR to assess the causal relationship of CHB, liver fibrosis and cirrhosis with T2D, aiming to reveal the love-hate relationship between HBV and T2D.

## 2 Materials and methods

### 2.1 Study design

**Figure 1:**
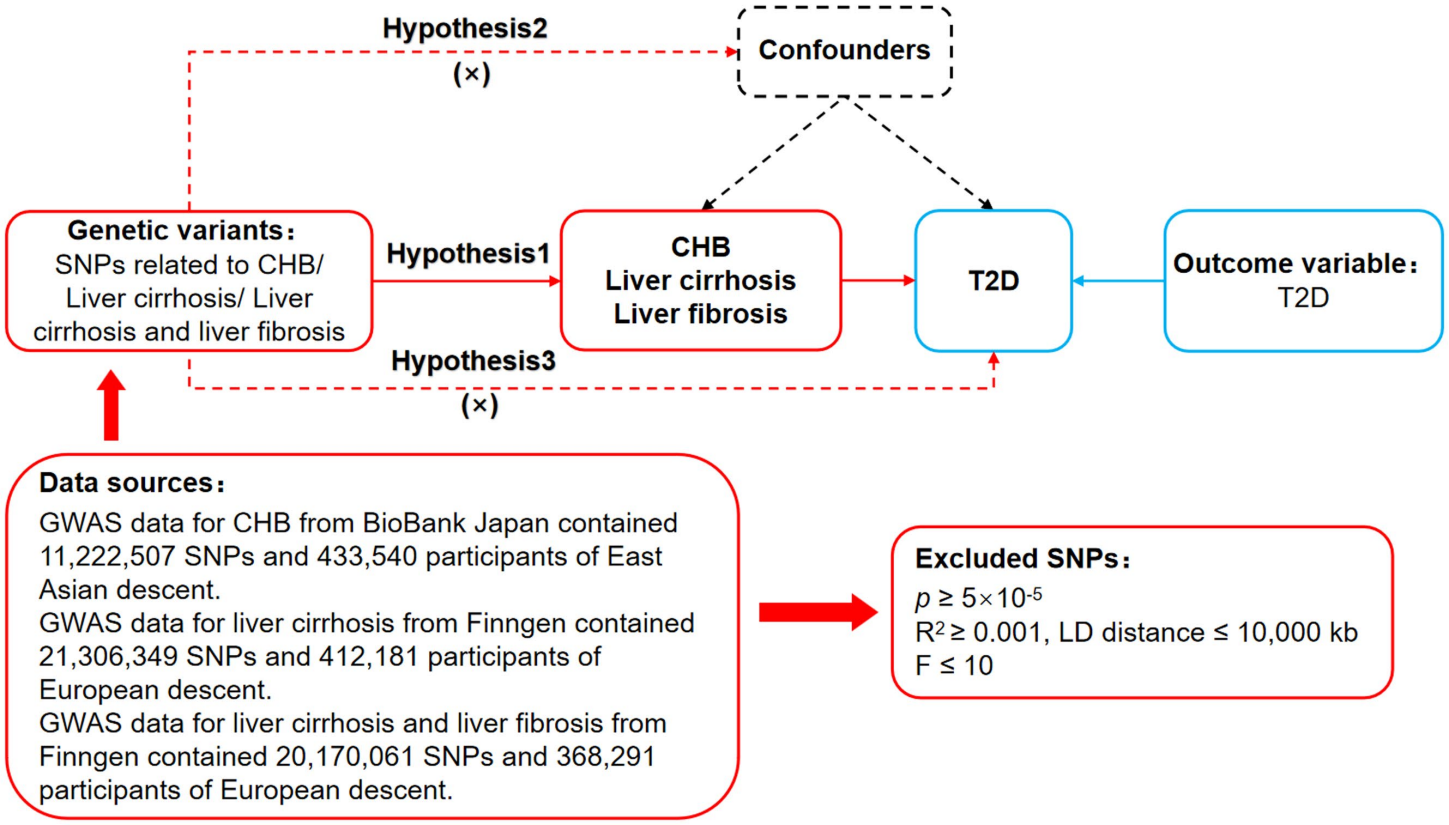
MR design for CHB and complications on genetic susceptibility to T2D. MR, Mendelian randomization; CHB, chronic hepatitis B; T2D, type 2 diabetes.

Mendelian randomization is based on three basic assumptions of association, independence, and exclusivity (Davies et al., 2018). The association assumption requires that single nucleotide polymorphisms (SNPs) are strongly correlated with exposure. The independence assumption requires that SNPs are independent of confounding factors. The exclusivity assumption requires that SNPs only act on outcome variables through exposure factor and not other pathways. The MR design process is shown in Figure 1.

### 2.2 Data sources

Data on CHB were obtained from BioBank Japan Project.^1^ Data on liver cirrhosis as well as liver cirrhosis and liver fibrosis were obtained from FinnGen.^2^ Data on T2D for East Asians was obtained from European Bioinformatics Institute,^3^ and data on T2D for Europeans was obtained from FinnGen (see text footnote 2). Since they were all derived from publicly available databases, the study did not require additional ethical approval.

### 2.3 Genetic instrumental variables

First, limiting *p* < 5 × 10^−5^ to search for SNPs closely associated with CHB, liver cirrhosis as well as liver cirrhosis and liver fibrosis in the dataset, to fulfill the association assumption. Second, limiting *R*^2^ < 0.001 and kb = 10,000 to search for SNPs with independence. Third, limiting *F* > 10 to search for SNPs with strong correlation. *F* = [ $R^{2}$ /( $1-R^{2}$ )]*[ (N−K−1 )/ k ]. *R*^2^ refers to the cumulative explained variance of the selected instrument variables on exposure, N refers to the sample size of the genome-wide association studies (GWAS), and K refers to number of paired samples. Fourth, the meanings of SNPs were searched through PhenoScanner, PubMed, and Web of Science, and SNPs that might be associated with T2D were excluded to fulfill the independence assumption. Fifth, mismatched SNPs were excluded based on the effect of allele frequency values when adjusting the allelic orientation of exposure and outcome. Sixth, SNPs with significant bias (*p* < 1) were excluded using the MR-Pleiotropy Residual Sum and Outlier method (MR-PRESSO) to ensure the correctness of causal inference.

### 2.4 Data analysis

The study followed the STROBE-MR guidelines (Skrivankova et al., 2021). R 4.3.1 software “TwoSampleMR (0.5.7)” was used for the MR analysis. Inverse variance weighted (IVW) was the main assessment tool, which allows for unbiased causal analysis without pleiotropy (Auton et al., 2015). Weighted median and MR-Egger were secondary assessment tools, with the former being less sensitive to error values and outliers, and the latter providing effective causal analysis in the presence of pleiotropy. MR-Egger intercept was used to assess horizontal pleiotropy, with *p* ≥ 0.05 indicating no significant pleiotropy to meet the exclusivity assumption. Cochran’s *Q* was used to assess heterogeneity, with *p* ≥ 0.05 indicating no significant heterogeneity. Leave-one-out sensitivity analysis was used to assess the robustness of the MR results, indicating that the results were robust when the combined effect sizes were all on the same side.

## 3 Results

### 3.1 GWAS data of the exposure

The CHB dataset (bbj-a-99) contains 212,453 East Asians and 11,074 closely related SNPs. The liver cirrhosis dataset (finngen_R10_CIRRHOSIS_BROAD) contains 321,192 Europeans and 2,310 closely related SNPs. The liver cirrhosis and liver fibrosis dataset (finngen_R9_K11_FIBROCHIRLIV) contained 368,291 Europeans and 2,569 closely related SNPs. Thirty-nine SNPs for CHB,105 SNPs for liver cirrhosis, and 78 SNPs for liver cirrhosis and liver fibrosis were included after independence testing and exclusivity testing, as shown in Supplementary Table S1. Mismatched and significantly biased SNPs were further excluded, and SNPs for final inclusion are shown in Supplementary Table S2.

### 3.2 GWAS data of the outcome

The East Asian T2D dataset (ebi-a-GCST010118) provides GWAS for 433,540 East Asians. The European T2D dataset (finngen_R9_T2D) provides GWAS for 377,277 Europeans. The exposure and outcome GWAS dataset are shown in Table 1.

**Table 1:** Details of the GWAS studies included in the Mendelian randomization.

Year	Trait	GWAS ID	Population	Sample size	Web source
2019	CHB	bbj-a-99	East Asian	212,453	https://biobankjp.org/en/
2023	Liver cirrhosis	finngen_R10_CIRRHOSIS_BROAD	European	321,192	www.finngen.fi/fi
2021	Liver cirrhosis and liver fibrosis	finngen_R9_K11_FIBROCHIRLIV	European	368,291	www.finngen.fi/fi
2020	T2D	ebi-a-GCST010118	East Asian	433,540	www.ebi.ac.uk
2021	T2D	finngen_R9_T2D	European	377,277	www.finngen.fi/fi

CHB, chronic hepatitis B; T2D, type 2 diabetes.

### 3.3 MR analysis results

**Figure 2:**
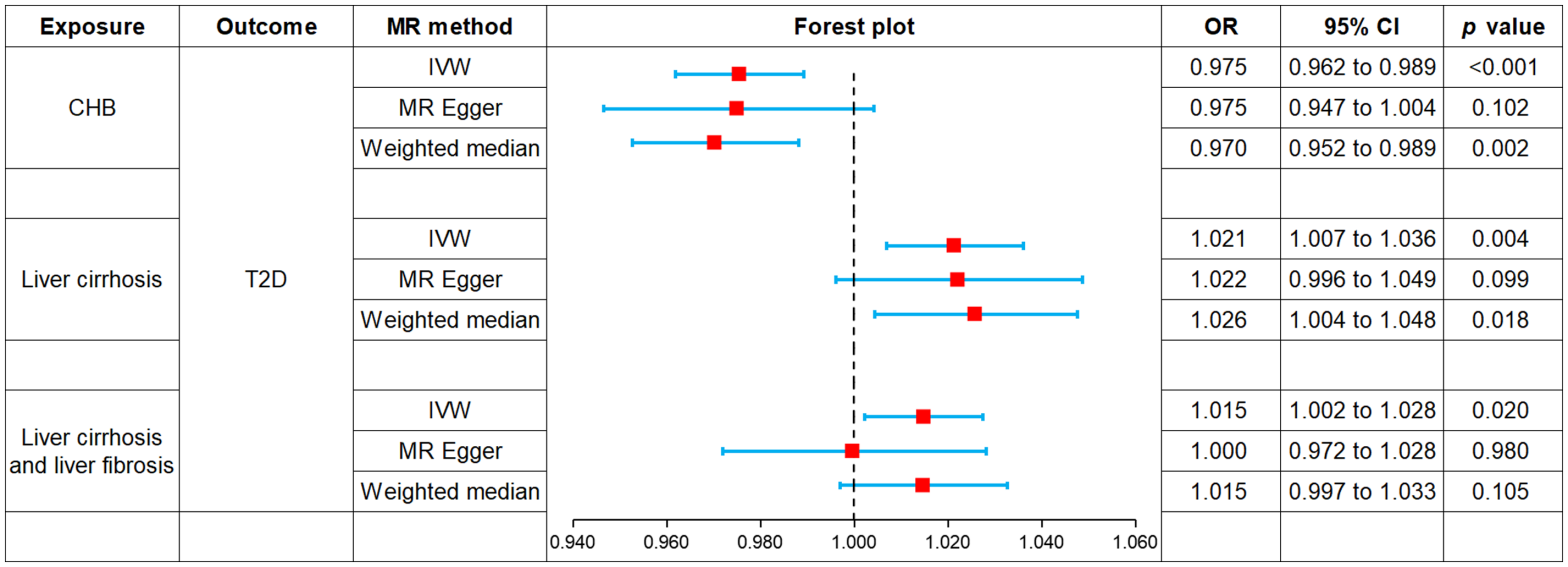
Forest plot of MR analysis for CHB and complications on genetic susceptibility to T2D. MR, Mendelian randomization; CHB, chronic hepatitis B; T2D, type 2 diabetes.

**Figure 3:**
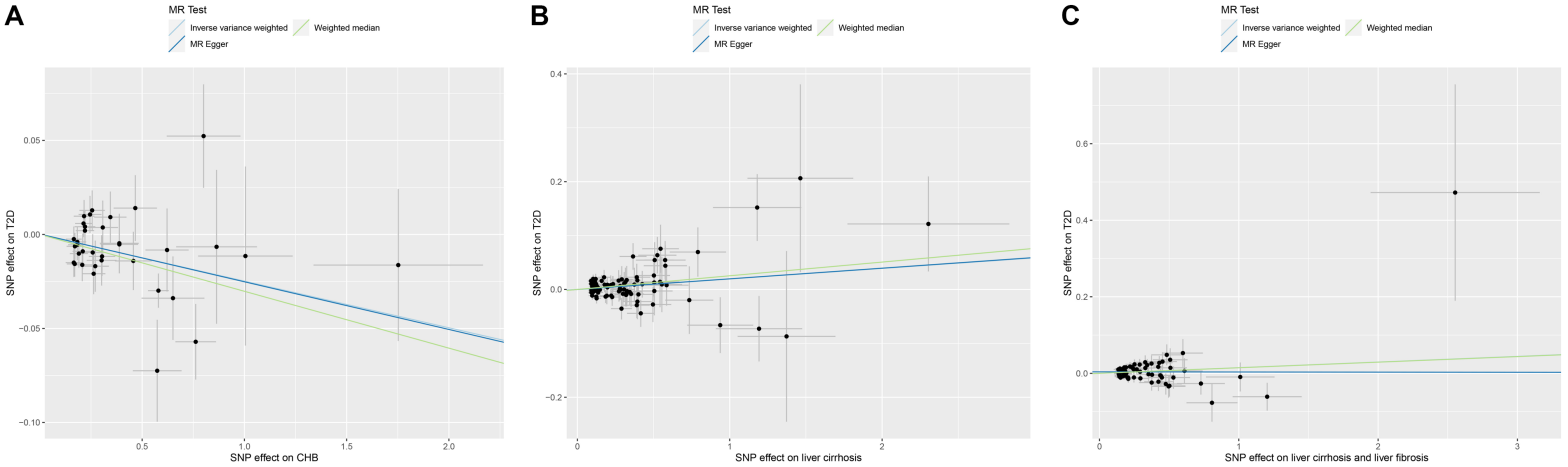
Scatter plot of MR analysis for CHB and complications on genetic susceptibility to T2D. **(A)** CHB on T2D. **(B)** Liver cirrhosis on T2D. **(C)** Liver cirrhosis and liver fibrosis on T2D. MR, Mendelian randomization; CHB, chronic hepatitis B; T2D, type 2 diabetes.

**Figure 4:**
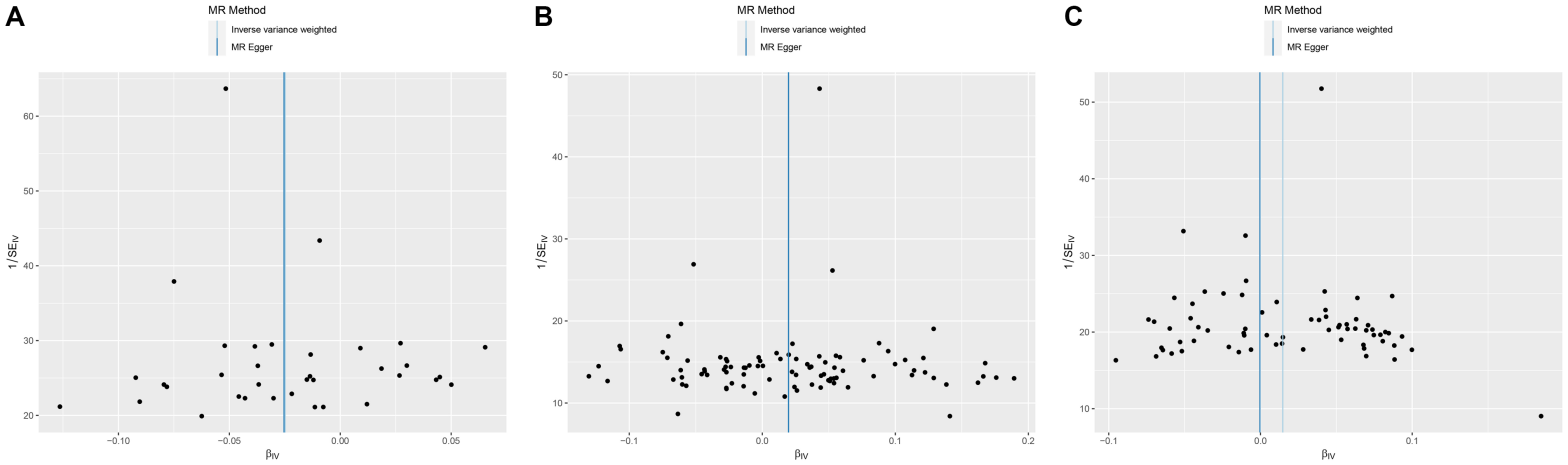
Funnel plot of MR analysis for CHB and complications on genetic susceptibility to T2D. **(A)** CHB on T2D. **(B)** Liver cirrhosis on T2D. **(C)** Liver cirrhosis and liver fibrosis on T2D. MR, Mendelian randomization; CHB, chronic hepatitis B; T2D, type 2 diabetes.

**Figure 5:**
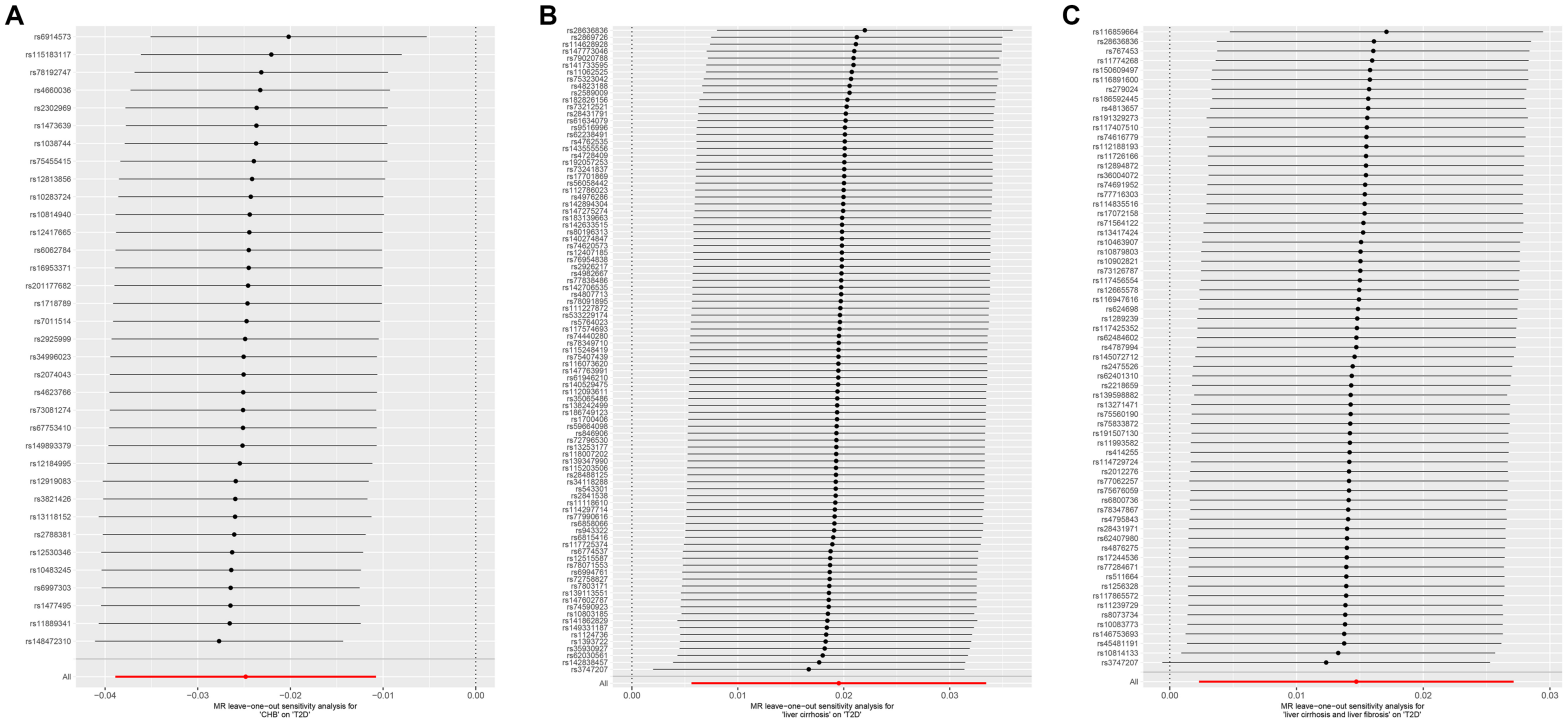
Leave-one-out sensitive analysis for CHB and complications on genetic susceptibility to T2D. **(A)** CHB on T2D. **(B)** Liver cirrhosis on T2D. **(C)** Liver cirrhosis and liver fibrosis on T2D. MR, Mendelian randomization; CHB, chronic hepatitis B; T2D, type 2 diabetes.

MR was employed to assess the causal effect between exposure and outcome, see Figure 2 for the forest plot and Figure 3 for the scatter plot. MR-Egger intercept analysis is shown in Supplementary Table S3. Cochran’s *Q* test is shown in Supplementary Table S4 and Figure 4. Leave-one-out sensitivity analysis is shown in Figure 5.

#### 3.3.1 Impact of CHB on T2D

IVW (OR, 0.975; 95% CI, 0.962–0.989, *p* < 0.001) and weighted median (OR, 0.970; 95% CI, 0.952–0.989; *p* = 0.002) showed that CHB was associated with a reduced genetic susceptibility to T2D, whereas MR-Egger (OR, 0.975; 95% CI, 0.947–1.004; *p =* 0.102) did not observe this effect. MR-Egger intercept showed no significant horizontal pleiotropy (*p* = 0.968). Cochran’s *Q* showed no heterogeneity (*p* = 0.06). Leave-one-out sensitivity analysis showed the results were robust.

#### 3.3.2 Impact of liver cirrhosis on T2D

IVW (OR, 1.021; 95% CI, 1.007–1.036; *p* = 0.004) and weighted median (OR, 1.026; 95% CI, 1.004–1.048; *p* = 0.018) showed that liver cirrhosis was associated with an increased genetic susceptibility to T2D, whereas MR-Egger (OR, 1.022; 95% CI, 0.996–1.049; *p* = 0.099) did not observe such an effect. MR-Egger intercept showed no significant horizontal pleiotropy (*p* = 0.944). Cochran’s *Q* showed no heterogeneity (*p* = 0.135). Leave-one-out sensitivity analysis showed the results were robust.

#### 3.3.3 Impact of liver cirrhosis and liver fibrosis on T2D

IVW showed that liver cirrhosis and liver fibrosis were associated with an increased genetic susceptibility to T2D (OR, 1.015; 95% CI, 1.002–1.028; *p* = 0.020), whereas MR-Egger (OR, 1.000; 95% CI, 0.972–1.028; *p* = 0.980) and weighted median (OR, 1.015; 95% CI, 0.997–1.033; *p* = 0.105) did not observe this effect. MR-Egger intercept showed no significant horizontal pleiotropy (*p* = 0.245). Cochran’s *Q* showed no heterogeneity (*p* = 0.076). Leave-one-out sensitivity analysis showed the results were robust.

## 4 Discussion

Type 2 diabetes remains a significant threat to human health and contributes to a reduced quality of life (Tinajero and Malik, 2021). Focusing on related risk factors, especially some chronic diseases, is relevant to the prevention and treatment of T2D (Yuan and Larsson, 2020). The relationship between HBV and T2D has been a topic of debate. On the one hand, HBV infection has been reported to be associated with an increased risk of diabetes mellitus (Cai et al., 2015), suggesting that it may be a potential risk factor for T2D. On the other hand, some other studies have instead suggested that HBV infection leads to a lower incidence of T2D (Li et al., 2016). To explore the complex and contradictory association between HBV and T2D, this study used MR to analyze the impact of HBV-related exposures (CHB, liver cirrhosis, as well as liver cirrhosis and liver fibrosis) on the genetic susceptibility to T2D.

This MR analysis showed that CHB was associated with a reduced genetic susceptibility to T2D, whereas liver fibrosis and cirrhosis were associated with an increased genetic susceptibility to T2D. These results were free of horizontal pleiotropy and heterogeneity and were robust. Notably, the data on CHB were from East Asians, and the data on liver fibrosis and cirrhosis were from Europeans. Therefore, the results primarily explain the role of CHB in reducing the risk of T2D in East Asians and the role of liver fibrosis and cirrhosis in increasing the risk of T2D in Europeans.

This study found that CHB was associated with a reduced risk of T2D in East Asians, in line with the clinical report conducted by Li et al. (2016). This cross-sectional study, which included 900 retired Chinese workers, revealed that a positive hepatitis B surface antibody (HBsAb) status was associated with a lower prevalence of diabetes after adjusting for factors such as gender, hypertension, dyslipidemia, family history of diabetes, and body mass index (OR, 0.579; 95% CI, 0.388–0.918) (Li et al., 2016). In addition to T2D, previous studies have observed a protective effects of HBV on other metabolic diseases such as metabolic syndrome (MS) and non-alcoholic fatty liver disease (NAFLD). In a cross-sectional study in Taiwan, Jan et al. (2006) reported that the incidence of MS was significantly lower in patients with positive hepatitis B surface antigen (HBsAg) compared to those without HBsAg (AOR, 0.84; 95% CI, 0.76–0.93). A clinical study conducted in Korea by Joo et al. (2017) showed that the HBsAg-positive group exhibited a lower incidence of NAFLD compared to the HBsAg-negative group (HR 0.83; 95% CI, 0.73–0.94). A cross-sectional study in Hong Kong observed similar results, indicating significantly lower prevalence rates of NAFLD (13.5% vs. 28.3%) and MS (11.0% vs. 20.2%) in individuals with HBV infection compared to the general population (Wong et al., 2012). A meta-analysis that included five studies and 119,903 subjects also showed that HBV-infected individuals had a 29% lower risk of NAFLD than non-HBV-infected individuals (OR, 0.71; 95% CI, 0.53–0.90) (Xiong et al., 2017). These pieces of evidence suggest that HBV has a role in reducing the risk of T2D, MS and NAFLD.

Considering that impaired glycolipid metabolism is a common pathogenesis of T2D, MS and NAFLD, the protective effect of HBV may be related to the regulation of glycolipid metabolism. For one thing, HBV reduces lipid levels and the risk of hepatic steatosis. A clinical study of HBV-infected patients without cirrhosis showed that in HBeAg-negative patients, high-load HBV infection was associated with a 26% reduced risk of hypertriglyceridemia (OR, 0.74; 95% CI, 0.61–0.89) (Chiang et al., 2013b). Another cohort study of middle-aged and elderly Taiwanese reported that patients with CHB had a lower risk of hepatic steatosis compared to non-infected individuals (OR, 0.54; 95% CI, 0.43–0.68) (Yu et al., 2022). For another, HBV promotes glucose metabolism by increasing adiponectin levels. It has been reported that chronically HBV-infected individuals have higher serum adiponectin levels, and these levels are positively correlated with HBV load in overweight or obese individuals (Chiang et al., 2013a). HBV replication increases the expression of peroxisome proliferator-activated receptor γ and its transcriptional activity in hepatocytes (Kim et al., 2007; Yoon et al., 2011), thereby promoting hepatic adiponectin production (Skat-Rørdam et al., 2019). Adiponectin has been shown to inhibit hepatic glucose synthesis and increase insulin sensitivity (Wang and Scherer, 2016). These pieces of evidence point to the fact that HBV has a function in regulating glucolipid metabolism, which may be an intrinsic mechanism by which CHB reduces the risk of T2D.

However, some other clinical studies have reported that CHB is not associated with T2D risk. A Taiwanese cohort study showed that the prevalence of diabetes mellitus in asymptomatic chronic HBV-infected individuals was comparable to that of uninfected individuals in 1997–1998 (9.49% vs. 12.0%) and 2000–2001 (11.2% vs. 13.0%) (Huang et al., 2010). Another cross-sectional study in Chinese Mainland showed that the serologic status of HBsAg and HBsAb was not associated with diabetes mellitus (Liu et al., 2020). Zhang et al. (2015) conducted a meta-analysis of 15 clinical studies and showed that the risk of T2D in patients with CHB was comparable to that of non-CHB patients (OR, 1.02; 95% CI, 0.78–1.34). These pieces of evidence support that CHB is not a protective factor for T2D, in contrast to the results of this MR analysis. Considering that clinical factors are a main cause of different results between MR analysis and clinical studies, we speculated that antiviral therapy may have contributed to the conflicting results. Previous studies have shown that high load HBV reduces the incidence of T2D by reducing the risk of hepatic steatosis (Yu et al., 2022). It suggests that a high viral load may be a critical factor of CHB that reduces the risk of T2D, not just HBsAb positivity. However, the reality is that, in order to manage their disease, most patients with CHB receive antiviral therapy, resulting in their HBV load being controlled at a relatively low level. Due to the reduced viral load, the regulatory role of HBV in glucose and lipid metabolism has been weakened, leading to its protective effect against T2D no longer being significant. This speculation is supported by Yu et al. (2022), whose cohort study found that functional cure of HBV infection significantly increased the risk of steatosis by 41% (OR, 1.41; 95% CI, 1.12–1.79). Additionally, they reported a significantly increased risk of progressive impairment of glucose metabolism due to steatosis after HBsAg seroclearance (Yu et al., 2022). In summary, CHB is a potential protective factor for T2D, but its role is limited by HBV load. Antiviral therapy may lead to a decrease in HBV load, which results in a weakening or even loss of the protective effect of HBV.

Although this study suggests that CHB is a protective factor for T2D, it does not apply to groups with concomitant liver fibrosis or cirrhosis. A meta-analysis demonstrated that patients with CHB cirrhosis had a 76% increased risk of T2D compared to patients with CHB without cirrhosis (OR, 1.76; 95% CI, 1.44–2.14) (Shen et al., 2017). Another meta-analysis indicated that patients with hepatitis B cirrhosis had an approximately 2-fold increased risk of T2D compared to non-HBV-infected patients (OR, 1.99; 95% CI, 1.08–3.65) (Zhang et al., 2015). Further meta-analysis found that the prevalence of diabetes in patients with hepatitis B cirrhosis, hepatitis C cirrhosis, cryptogenic cirrhosis, alcoholic cirrhosis, and NAFLD cirrhosis were 22.2, 32.2, 50.8, 27.3, and 56.1%, respectively, suggesting that cirrhosis due to different etiologies may increase the risk of diabetes (Lee et al., 2019). These pieces of evidence support that cirrhosis is a potential risk factor for T2D, supporting the results of this MR analysis. Indeed, since chronic HBV infection continues to mediate immune damage in the liver, poorly controlled CHB eventually progresses to liver fibrosis and even cirrhosis (Bedossa, 2015; Seeger and Mason, 2015). And cirrhosis leads to impaired insulin clearance from the liver, resulting in pancreatic β-cell dysfunction and peripheral insulin resistance (Kingston et al., 1984; Grancini et al., 2015; Elkrief et al., 2016), consequently increasing the risk of T2D. It is important to note that globally, approximately 2–4% of HBV-infected individuals develop cirrhosis each year (Zhao et al., 2020), which suggests that the majority of CHB patients do not have comorbid cirrhosis. Due to the absolute proportion of CHB patients receiving antiviral treatment and not suffering from liver cirrhosis, published clinical studies on non-specific CHB groups have not found a causal relationship with T2D. In conclusion, CHB is not an absolute protective factor for T2D. When it progresses to liver fibrosis or cirrhosis, it reverses and becomes a risk factor for T2D.

To explore the genetic mechanisms underlying the impact of CHB, liver fibrosis and cirrhosis on T2D risk, we searched for the included SNPs in public databases. Supplementary Table S5 shows six SNPs (rs10483245, rs1256328, rs11889341, rs846906, rs6858066 and rs3747207) that have been cited in publications. Among them, only rs846906-T has been reported to be significantly associated with increased waist circumference, triglycerides and MS risk (OR 3.31, 95% CI 1.53–7.17) (Quteineh et al., 2015), while rs10483245, rs1256328, rs11889341, rs6858066 and rs3747207 have no direct or indirect relationship with T2D. The remaining SNPs have not been cited in any publications, suggesting their role or importance in health and disease may not yet be established. Further research is needed to elucidate the significance of these SNPs in the development of T2D.

Undeniably, there are some limitations to this MR analysis. First, because the dataset was derived from Europeans and East Asians, the study mainly revealed the effect of CHB on genetic susceptibility to T2D in East Asians and the effect of liver fibrosis and cirrhosis on genetic susceptibility to T2D in Europeans, caution is needed when used for other ethnicities. Second, because CHB was derived from pooled data from GWAS, it was not possible to stratify the analysis for groups with different viral loads and comorbidities. Third, due to the lack of data on hepatitis B-related liver fibrosis and cirrhosis in GWAS, this study utilized data on non-specific liver fibrosis and cirrhosis, which may limit its ability to fully elucidate the impact of hepatitis B-related liver fibrosis and cirrhosis on the risk of T2D. Given these limitations, future studies should continue to focus on human genome-wide studies, providing more comprehensive data to advance MR analysis across different ethnicities. Meanwhile, high-quality stratified clinical studies need to be continued for exploring the impact of different viral loads and different stages of HBV infection on T2D risk. We look forward to more evidence revealing the love-hate relationship between HBV and T2D.

## 5 Conclusion

CHB has the potential to act as a protective factor for T2D, but its effectiveness is constrained by viral load and disease stage. This protective effect diminishes or disappears as viral load decreases, and it transforms into a risk factor with the progression to liver fibrosis and cirrhosis.

## Data availability statement

The original contributions presented in the study are included in the article/Supplementary material, further inquiries can be directed to the corresponding author.

## Ethics statement

This study is based on published experimental research and is not currently applicable to medical ethics.

## Author contributions

YY: Conceptualization, Supervision, Writing – original draft. KT: Formal analysis, Writing – original draft. GH: Methodology, Writing – original draft. XY: Data curation, Writing – original draft. JW: Data curation, Writing – original draft. SB: Formal analysis, Writing – original draft. RY: Conceptualization, Supervision, Writing – review & editing.

## Footnotes

^1^https://biobankjp.org/en/

^2^www.finngen.fi/fi

^3^https://www.ebi.ac.uk
