# Supplementary material for: Love-hate relationship between hepatitis B virus and type 2 diabetes: a Mendelian randomization study
Source: Front Microbiol. 2024 Apr 5;15:1378311. doi: 10.3389/fmicb.2024.1378311 (PMC11026703; doi:10.3389/fmicb.2024.1378311)
Supplement: Supplementary file 1 [file Table_1.DOCX]

**Supplementary Table S1.1** Characteristics of instrumental variables for CHB

|  | **SNP** | **EA** | **OA** | **Samplesize** | **SE** | **β** | **id.exposure** | **EAF** | ***p* value** | **R^2^** | **F - statistic** |
| --- | --- | --- | --- | --- | --- | --- | --- | --- | --- | --- | --- |
| 1 | rs4623766 | A | T | 212453 | 0.0917267 | 0.388497 | bbj-a-99 | 0.0495036 | 2.28E-05 | 0.014203409 | 3061.005159 |
| 2 | rs1038744 | A | G | 212453 | 0.0496923 | -0.207233 | bbj-a-99 | 0.820147 | 3.04E-05 | 0.012669434 | 2726.172975 |
| 3 | rs4660036 | A | G | 212453 | 0.0382738 | 0.170285 | bbj-a-99 | 0.548546 | 8.62E-06 | 0.014361815 | 3095.641074 |
| 4 | rs67753410 | T | C | 212453 | 0.0950659 | 0.388416 | bbj-a-99 | 0.0545363 | 4.39E-05 | 0.015558035 | 3357.557114 |
| 5 | rs11889341 | T | C | 212453 | 0.0413158 | -0.213492 | bbj-a-99 | 0.306142 | 2.37E-07 | 0.019363628 | 4195.053505 |
| 6 | rs3821426 | G | C | 212453 | 0.0506994 | 0.220563 | bbj-a-99 | 0.199991 | 1.36E-05 | 0.015566846 | 3359.488735 |
| 7 | rs201177682 | C | A | 212453 | 0.0407488 | 0.171378 | bbj-a-99 | 0.421182 | 2.60E-05 | 0.014320295 | 3086.561481 |
| 8 | rs2622605 | C | T | 212453 | 0.044004 | -0.193383 | bbj-a-99 | 0.752947 | 1.11E-05 | 0.013913011 | 2997.537828 |
| 9 | rs13118152 | A | G | 212453 | 0.414197 | 1.75228 | bbj-a-99 | 0.00363733 | 2.33E-05 | 0.02225549 | 4835.824705 |
| 10 | rs1718789 | G | T | 212453 | 0.0898452 | 0.456981 | bbj-a-99 | 0.0499333 | 3.65E-07 | 0.019813931 | 4294.582024 |
| 11 | rs12530346 | G | A | 212453 | 0.105879 | 0.466509 | bbj-a-99 | 0.0350716 | 1.05E-05 | 0.014729931 | 3176.17342 |
| 12 | rs6914573 | T | A | 212453 | 0.0505987 | 0.579461 | bbj-a-99 | 0.754931 | 2.30E-30 | 0.124243609 | 30140.43534 |
| 13 | rs16870693 | A | C | 212453 | 0.0524686 | 0.3457 | bbj-a-99 | 0.170368 | 4.44E-11 | 0.033783316 | 7428.25018 |
| 14 | rs115183117 | C | G | 212453 | 0.0994431 | -0.76187 | bbj-a-99 | 0.0461655 | 1.84E-14 | 0.051118996 | 11445.35687 |
| 15 | rs77746323 | T | C | 212453 | 0.0397601 | 0.516645 | bbj-a-99 | 0.598984 | 1.32E-38 | 0.128230513 | 31249.89015 |
| 16 | rs149893379 | A | C | 212453 | 0.105466 | -0.621793 | bbj-a-99 | 0.0387667 | 3.73E-09 | 0.028814381 | 6303.26884 |
| 17 | rs78192747 | T | A | 212453 | 0.11913 | -0.573659 | bbj-a-99 | 0.0343878 | 1.47E-06 | 0.021854695 | 4746.791545 |
| 18 | rs2788381 | A | C | 212453 | 0.0788673 | -0.344524 | bbj-a-99 | 0.937221 | 1.25E-05 | 0.013967715 | 3009.490707 |
| 19 | rs1477495 | A | C | 212453 | 0.0505638 | 0.216089 | bbj-a-99 | 0.827479 | 1.92E-05 | 0.013331968 | 2870.661503 |
| 20 | rs7011514 | C | A | 212453 | 0.0447543 | 0.182822 | bbj-a-99 | 0.238804 | 4.41E-05 | 0.012151363 | 2613.324718 |
| 21 | rs34996023 | A | G | 212453 | 0.231217 | 1.00498 | bbj-a-99 | 0.0110667 | 1.38E-05 | 0.022107008 | 4802.832244 |
| 22 | rs6997303 | T | C | 212453 | 0.0620755 | 0.255547 | bbj-a-99 | 0.110229 | 3.84E-05 | 0.012809898 | 2756.789915 |
| 23 | rs10814940 | G | C | 212453 | 0.0646476 | 0.303951 | bbj-a-99 | 0.104695 | 2.58E-06 | 0.01731945 | 3744.385195 |
| 24 | rs10283724 | T | C | 212453 | 0.0664273 | -0.27063 | bbj-a-99 | 0.90662 | 4.62E-05 | 0.012401124 | 2667.713756 |
| 25 | rs11139174 | G | A | 212453 | 0.0450695 | 0.224809 | bbj-a-99 | 0.240355 | 6.10E-07 | 0.018455305 | 3994.569001 |
| 26 | rs148472310 | T | G | 212453 | 0.180346 | 0.800492 | bbj-a-99 | 0.0129801 | 9.05E-06 | 0.016419047 | 3546.472563 |
| 27 | rs2074043 | A | G | 212453 | 0.0386206 | 0.166053 | bbj-a-99 | 0.405042 | 1.71E-05 | 0.013289536 | 2861.4019 |
| 28 | rs12417665 | T | C | 212453 | 0.0727445 | -0.301759 | bbj-a-99 | 0.0783586 | 3.35E-05 | 0.013152221 | 2831.442288 |
| 29 | rs73081274 | C | A | 212453 | 0.197656 | 0.863862 | bbj-a-99 | 0.0129086 | 1.24E-05 | 0.01901758 | 4118.630234 |
| 30 | rs12813856 | T | C | 212453 | 0.044127 | 0.190644 | bbj-a-99 | 0.309394 | 1.56E-05 | 0.015531683 | 3351.780338 |
| 31 | rs1473639 | T | C | 212453 | 0.0398703 | 0.165926 | bbj-a-99 | 0.354561 | 3.16E-05 | 0.012601001 | 2711.259879 |
| 32 | rs12184995 | G | A | 212453 | 0.0745566 | -0.307495 | bbj-a-99 | 0.922631 | 3.72E-05 | 0.013498986 | 2907.116139 |
| 33 | rs75455415 | T | C | 212453 | 0.153755 | 0.650728 | bbj-a-99 | 0.0183803 | 2.31E-05 | 0.015280052 | 3296.635185 |
| 34 | rs12919083 | C | A | 212453 | 0.0451168 | 0.220315 | bbj-a-99 | 0.23608 | 1.04E-06 | 0.017507543 | 3785.774669 |
| 35 | rs16953371 | T | C | 212453 | 0.0528411 | 0.258305 | bbj-a-99 | 0.162354 | 1.02E-06 | 0.018147597 | 3926.736016 |
| 36 | rs2925999 | C | T | 212453 | 0.0434623 | 0.183066 | bbj-a-99 | 0.263295 | 2.53E-05 | 0.013001145 | 2798.489878 |
| 37 | rs2302969 | A | G | 212453 | 0.057438 | -0.262897 | bbj-a-99 | 0.128256 | 4.72E-06 | 0.015454961 | 3334.963659 |
| 38 | rs6062784 | A | G | 212453 | 0.0497503 | 0.209591 | bbj-a-99 | 0.246357 | 2.52E-05 | 0.016311948 | 3522.955967 |
| 39 | rs10483245 | T | A | 212453 | 0.0559042 | -0.24512 | bbj-a-99 | 0.148707 | 1.16E-05 | 0.015212408 | 3281.815621 |

SNP, single nucleotide polymorphism; EA, effect allele; OA, other allele; EAF, effect allele frequency; SE, standard error; CHB, chronic hepatitis B.

**Supplementary Table S1.2** Characteristics of instrumental variables for liver cirrhosis

|  | **SNP** | **EA** | **OA** | **Samplesize** | **SE** | **β** | **id.exposure** | **EAF** | ***p* value** | **R^2^** | **F - statistic** |
| --- | --- | --- | --- | --- | --- | --- | --- | --- | --- | --- | --- |
| 1 | rs12141891 | C | A | 412181 | 0.0261759 | -0.11324 | finngen_R10_CIRRHOSIS_BROAD | 0.228029 | 1.52E-05 | 0.004514775 | 1869.335135 |
| 2 | rs115248419 | G | A | 412181 | 0.0593561 | 0.281388 | finngen_R10_CIRRHOSIS_BROAD | 0.028704 | 2.13E-06 | 0.004415045 | 1827.859031 |
| 3 | rs114628928 | G | A | 412181 | 0.0904261 | -0.41622 | finngen_R10_CIRRHOSIS_BROAD | 0.019446 | 4.17E-06 | 0.006606627 | 2741.223031 |
| 4 | rs141862829 | A | G | 412181 | 0.53116 | -2.30384 | finngen_R10_CIRRHOSIS_BROAD | 0.00158365 | 1.44E-05 | 0.016784388 | 7036.271834 |
| 5 | rs846906 | C | T | 412181 | 0.0297206 | 0.123307 | finngen_R10_CIRRHOSIS_BROAD | 0.830927 | 3.34E-05 | 0.004272112 | 1768.429615 |
| 6 | rs11118610 | C | A | 412181 | 0.0215647 | 0.097829 | finngen_R10_CIRRHOSIS_BROAD | 0.471064 | 5.72E-06 | 0.004769269 | 1975.212845 |
| 7 | rs75407439 | T | A | 412181 | 0.0872245 | 0.374057 | finngen_R10_CIRRHOSIS_BROAD | 0.0123627 | 1.80E-05 | 0.003416775 | 1413.151329 |
| 8 | rs12407185 | T | C | 412181 | 0.0785094 | -0.32599 | finngen_R10_CIRRHOSIS_BROAD | 0.0237557 | 3.29E-05 | 0.004928918 | 2041.659568 |
| 9 | rs72758827 | A | T | 412181 | 0.0408777 | 0.175432 | finngen_R10_CIRRHOSIS_BROAD | 0.0687549 | 1.77E-05 | 0.00394108 | 1630.857865 |
| 10 | rs10803185 | T | C | 412181 | 0.0296457 | 0.120244 | finngen_R10_CIRRHOSIS_BROAD | 0.148519 | 4.99E-05 | 0.003656906 | 1512.832166 |
| 11 | rs61634079 | G | A | 412181 | 0.022194 | 0.091895 | finngen_R10_CIRRHOSIS_BROAD | 0.550254 | 3.46E-05 | 0.004179674 | 1730.004616 |
| 12 | rs79020788 | T | C | 412181 | 0.0690246 | -0.28958 | finngen_R10_CIRRHOSIS_BROAD | 0.0301747 | 2.73E-05 | 0.00490782 | 2032.877195 |
| 13 | rs72796530 | T | C | 412181 | 0.042136 | 0.178628 | finngen_R10_CIRRHOSIS_BROAD | 0.064069 | 2.24E-05 | 0.003826669 | 1583.331292 |
| 14 | rs74590923 | A | G | 412181 | 0.184783 | -0.79096 | finngen_R10_CIRRHOSIS_BROAD | 0.00598837 | 1.86E-05 | 0.007448047 | 3092.965226 |
| 15 | rs78091895 | G | A | 412181 | 0.0730063 | -0.30645 | finngen_R10_CIRRHOSIS_BROAD | 0.0271983 | 2.70E-05 | 0.0049694 | 2058.512056 |
| 16 | rs77990616 | G | A | 412181 | 0.346933 | 1.46343 | finngen_R10_CIRRHOSIS_BROAD | 0.00045464 | 2.46E-05 | 0.001946454 | 803.8519557 |
| 17 | rs141733595 | G | A | 412181 | 0.216437 | -0.93759 | finngen_R10_CIRRHOSIS_BROAD | 0.00453711 | 1.48E-05 | 0.007940643 | 3299.163841 |
| 18 | rs112786023 | T | C | 412181 | 0.0783249 | -0.32184 | finngen_R10_CIRRHOSIS_BROAD | 0.0245693 | 3.97E-05 | 0.00496474 | 2056.572054 |
| 19 | rs75323042 | C | T | 412181 | 0.085486 | -0.3898 | finngen_R10_CIRRHOSIS_BROAD | 0.0206815 | 5.12E-06 | 0.006155007 | 2552.676491 |
| 20 | rs142838457 | A | C | 412181 | 0.289682 | -1.18018 | finngen_R10_CIRRHOSIS_BROAD | 0.003158 | 4.62E-05 | 0.0087693 | 3646.498736 |
| 21 | rs6774537 | T | C | 412181 | 0.0215797 | -0.09168 | finngen_R10_CIRRHOSIS_BROAD | 0.542527 | 2.15E-05 | 0.0041724 | 1726.981266 |
| 22 | rs114297714 | T | C | 412181 | 0.0580439 | 0.270902 | finngen_R10_CIRRHOSIS_BROAD | 0.0313593 | 3.05E-06 | 0.004458446 | 1845.90761 |
| 23 | rs138242499 | T | C | 412181 | 0.0622397 | 0.307225 | finngen_R10_CIRRHOSIS_BROAD | 0.0263938 | 7.97E-07 | 0.004850967 | 2009.213511 |
| 24 | rs149331187 | T | C | 412181 | 0.125253 | -0.52507 | finngen_R10_CIRRHOSIS_BROAD | 0.0108512 | 2.76E-05 | 0.005918303 | 2453.923218 |
| 25 | rs78349710 | C | T | 412181 | 0.0413737 | -0.19714 | finngen_R10_CIRRHOSIS_BROAD | 0.0852538 | 1.89E-06 | 0.006061938 | 2513.84226 |
| 26 | rs59664098 | A | G | 412181 | 0.0441348 | 0.196382 | finngen_R10_CIRRHOSIS_BROAD | 0.0576132 | 8.60E-06 | 0.004187787 | 1733.376714 |
| 27 | rs73241837 | A | T | 412181 | 0.0219895 | -0.10613 | finngen_R10_CIRRHOSIS_BROAD | 0.414638 | 1.39E-06 | 0.005467847 | 2266.122339 |
| 28 | rs28488125 | T | C | 412181 | 0.0297561 | 0.125997 | finngen_R10_CIRRHOSIS_BROAD | 0.144747 | 2.29E-05 | 0.003930561 | 1626.487869 |
| 29 | rs6858066 | G | A | 412181 | 0.0217149 | 0.108233 | finngen_R10_CIRRHOSIS_BROAD | 0.524537 | 6.22E-07 | 0.005843086 | 2422.552327 |
| 30 | rs77833957 | T | C | 412181 | 0.0542713 | -0.25128 | finngen_R10_CIRRHOSIS_BROAD | 0.0480139 | 3.66E-06 | 0.005772181 | 2392.98473 |
| 31 | rs28636836 | T | C | 412181 | 0.0271778 | -0.22203 | finngen_R10_CIRRHOSIS_BROAD | 0.215987 | 3.10E-16 | 0.016695533 | 6998.389772 |
| 32 | rs2869726 | G | A | 412181 | 0.0228758 | -0.1246 | finngen_R10_CIRRHOSIS_BROAD | 0.684804 | 5.13E-08 | 0.006701918 | 2781.028173 |
| 33 | rs6815416 | T | C | 412181 | 0.0298633 | -0.12214 | finngen_R10_CIRRHOSIS_BROAD | 0.16316 | 4.32E-05 | 0.004073688 | 1685.956582 |
| 34 | rs77838486 | T | A | 412181 | 0.0780984 | 0.324516 | finngen_R10_CIRRHOSIS_BROAD | 0.0161704 | 3.25E-05 | 0.003350757 | 1385.7548 |
| 35 | rs78071553 | T | C | 412181 | 0.120119 | 0.545978 | finngen_R10_CIRRHOSIS_BROAD | 0.00595842 | 5.49E-06 | 0.003531148 | 1460.622813 |
| 36 | rs142633515 | T | G | 412181 | 0.0560548 | 0.272993 | finngen_R10_CIRRHOSIS_BROAD | 0.0334504 | 1.12E-06 | 0.004819017 | 1995.916055 |
| 37 | rs115203506 | A | G | 412181 | 0.0500996 | -0.23395 | finngen_R10_CIRRHOSIS_BROAD | 0.0566803 | 3.02E-06 | 0.005852746 | 2426.581364 |
| 38 | rs10043068 | A | G | 412181 | 0.0272139 | 0.113794 | finngen_R10_CIRRHOSIS_BROAD | 0.184798 | 2.90E-05 | 0.003901497 | 1614.413572 |
| 39 | rs116073620 | C | T | 412181 | 0.127068 | 0.542646 | finngen_R10_CIRRHOSIS_BROAD | 0.00524145 | 1.95E-05 | 0.003070664 | 1269.561726 |
| 40 | rs35065486 | A | G | 412181 | 0.0245022 | -0.11227 | finngen_R10_CIRRHOSIS_BROAD | 0.277596 | 4.61E-06 | 0.005055075 | 2094.181876 |
| 41 | rs186749123 | C | T | 412181 | 0.0771106 | 0.315216 | finngen_R10_CIRRHOSIS_BROAD | 0.0164468 | 4.35E-05 | 0.003214591 | 1329.26007 |
| 42 | rs4976286 | A | C | 412181 | 0.0306841 | -0.13265 | finngen_R10_CIRRHOSIS_BROAD | 0.155427 | 1.54E-05 | 0.004619501 | 1912.89794 |
| 43 | rs12515587 | G | T | 412181 | 0.0356271 | -0.15952 | finngen_R10_CIRRHOSIS_BROAD | 0.113097 | 7.55E-06 | 0.005105031 | 2114.983595 |
| 44 | rs2841538 | A | G | 412181 | 0.0715776 | 0.291609 | finngen_R10_CIRRHOSIS_BROAD | 0.0217082 | 4.62E-05 | 0.003611803 | 1494.105819 |
| 45 | rs74620573 | A | C | 412181 | 0.157809 | 0.734516 | finngen_R10_CIRRHOSIS_BROAD | 0.00306459 | 3.25E-06 | 0.003296643 | 1363.301332 |
| 46 | rs140529475 | T | A | 412181 | 0.121708 | -0.50435 | finngen_R10_CIRRHOSIS_BROAD | 0.0109256 | 3.41E-05 | 0.005497561 | 2278.505254 |
| 47 | rs147275274 | A | G | 412181 | 0.0656159 | 0.316295 | finngen_R10_CIRRHOSIS_BROAD | 0.0232158 | 1.43E-06 | 0.004537294 | 1878.701559 |
| 48 | rs143555556 | G | C | 412181 | 0.0463523 | 0.193489 | finngen_R10_CIRRHOSIS_BROAD | 0.0514244 | 2.99E-05 | 0.003652445 | 1510.980085 |
| 49 | rs147763991 | G | A | 412181 | 0.0887322 | -0.42574 | finngen_R10_CIRRHOSIS_BROAD | 0.0198493 | 1.60E-06 | 0.007052791 | 2927.660679 |
| 50 | rs139113551 | T | C | 412181 | 0.134299 | -0.57608 | finngen_R10_CIRRHOSIS_BROAD | 0.00994173 | 1.79E-05 | 0.00653304 | 2710.489498 |
| 51 | rs192057253 | A | C | 412181 | 0.0935595 | 0.394037 | finngen_R10_CIRRHOSIS_BROAD | 0.0110338 | 2.54E-05 | 0.003388524 | 1401.427184 |
| 52 | rs118007202 | C | T | 412181 | 0.07444 | 0.324092 | finngen_R10_CIRRHOSIS_BROAD | 0.0173718 | 1.34E-05 | 0.003585921 | 1483.360354 |
| 53 | rs35930927 | T | C | 412181 | 0.0248473 | 0.103859 | finngen_R10_CIRRHOSIS_BROAD | 0.243063 | 2.92E-05 | 0.003969144 | 1642.5172 |
| 54 | rs7803171 | A | G | 412181 | 0.0270287 | -0.11507 | finngen_R10_CIRRHOSIS_BROAD | 0.211929 | 2.07E-05 | 0.004422698 | 1831.041495 |
| 55 | rs4728409 | C | T | 412181 | 0.0235203 | 0.102165 | finngen_R10_CIRRHOSIS_BROAD | 0.281662 | 1.40E-05 | 0.004223684 | 1748.297927 |
| 56 | rs142706535 | C | A | 412181 | 0.0639196 | -0.28308 | finngen_R10_CIRRHOSIS_BROAD | 0.0355065 | 9.48E-06 | 0.005488679 | 2274.803786 |
| 57 | rs13253177 | T | G | 412181 | 0.0307817 | 0.127324 | finngen_R10_CIRRHOSIS_BROAD | 0.134751 | 3.53E-05 | 0.003780277 | 1564.063467 |
| 58 | rs73212521 | G | C | 412181 | 0.0551774 | -0.2285 | finngen_R10_CIRRHOSIS_BROAD | 0.0465316 | 3.45E-05 | 0.004632981 | 1918.505774 |
| 59 | rs6994761 | G | A | 412181 | 0.0217344 | 0.096509 | finngen_R10_CIRRHOSIS_BROAD | 0.424715 | 8.98E-06 | 0.004551432 | 1884.582318 |
| 60 | rs139347990 | T | C | 412181 | 0.105776 | 0.502305 | finngen_R10_CIRRHOSIS_BROAD | 0.00769984 | 2.05E-06 | 0.00385558 | 1595.340223 |
| 61 | rs2926217 | C | T | 412181 | 0.0215059 | 0.091982 | finngen_R10_CIRRHOSIS_BROAD | 0.472597 | 1.89E-05 | 0.004217647 | 1745.788501 |
| 62 | rs56058442 | T | C | 412181 | 0.0230186 | 0.111396 | finngen_R10_CIRRHOSIS_BROAD | 0.321232 | 1.30E-06 | 0.005411396 | 2242.59956 |
| 63 | rs80196313 | C | T | 412181 | 0.0914436 | 0.400915 | finngen_R10_CIRRHOSIS_BROAD | 0.0113427 | 1.16E-05 | 0.00360493 | 1491.25228 |
| 64 | rs115346713 | A | G | 412181 | 0.0607036 | 0.252934 | finngen_R10_CIRRHOSIS_BROAD | 0.0285354 | 3.09E-05 | 0.003546952 | 1467.183337 |
| 65 | rs147773046 | T | C | 412181 | 0.283089 | -1.19233 | finngen_R10_CIRRHOSIS_BROAD | 0.00292601 | 2.53E-05 | 0.008295186 | 3447.700814 |
| 66 | rs140274847 | T | C | 412181 | 0.0725626 | 0.298939 | finngen_R10_CIRRHOSIS_BROAD | 0.0192344 | 3.79E-05 | 0.003371623 | 1394.413668 |
| 67 | rs34118288 | A | T | 412181 | 0.0239236 | -0.10039 | finngen_R10_CIRRHOSIS_BROAD | 0.726162 | 2.72E-05 | 0.004007697 | 1658.535433 |
| 68 | rs74440280 | A | C | 412181 | 0.132966 | 0.554716 | finngen_R10_CIRRHOSIS_BROAD | 0.00445267 | 3.02E-05 | 0.002728059 | 1127.524698 |
| 69 | rs140201358 | G | C | 412181 | 0.0834209 | 0.370738 | finngen_R10_CIRRHOSIS_BROAD | 0.0138549 | 8.82E-06 | 0.003755852 | 1553.919424 |
| 70 | rs76954838 | T | C | 412181 | 0.320979 | 1.37282 | finngen_R10_CIRRHOSIS_BROAD | 0.000560652 | 1.89E-05 | 0.002112064 | 872.3908445 |
| 71 | rs142548379 | G | C | 412181 | 0.0478722 | 0.252622 | finngen_R10_CIRRHOSIS_BROAD | 0.0454363 | 1.31E-07 | 0.005535798 | 2294.441103 |
| 72 | rs11021222 | T | G | 412181 | 0.233386 | 1.10464 | finngen_R10_CIRRHOSIS_BROAD | 0.00113147 | 2.21E-06 | 0.002758182 | 1140.009 |
| 73 | rs111227872 | A | G | 412181 | 0.11988 | 0.50509 | finngen_R10_CIRRHOSIS_BROAD | 0.00580859 | 2.52E-05 | 0.002946512 | 1218.0796 |
| 74 | rs11062525 | T | C | 412181 | 0.0293752 | 0.1264 | finngen_R10_CIRRHOSIS_BROAD | 0.151662 | 1.69E-05 | 0.004111212 | 1701.550573 |
| 75 | rs182826156 | A | C | 412181 | 0.120143 | -0.49603 | finngen_R10_CIRRHOSIS_BROAD | 0.0113247 | 3.65E-05 | 0.005509768 | 2283.592545 |
| 76 | rs117725374 | G | C | 412181 | 0.141748 | -0.57749 | finngen_R10_CIRRHOSIS_BROAD | 0.00848079 | 4.62E-05 | 0.005608547 | 2324.763811 |
| 77 | rs4762535 | G | C | 412181 | 0.0220369 | -0.0961 | finngen_R10_CIRRHOSIS_BROAD | 0.405446 | 1.30E-05 | 0.004452128 | 1843.280237 |
| 78 | rs147602787 | G | A | 412181 | 0.123035 | -0.5075 | finngen_R10_CIRRHOSIS_BROAD | 0.0107499 | 3.71E-05 | 0.005477881 | 2270.304086 |
| 79 | rs1700406 | A | T | 412181 | 0.0540629 | -0.23187 | finngen_R10_CIRRHOSIS_BROAD | 0.0478132 | 1.80E-05 | 0.004895579 | 2027.782061 |
| 80 | rs61946210 | T | C | 412181 | 0.0326558 | -0.13478 | finngen_R10_CIRRHOSIS_BROAD | 0.141664 | 3.67E-05 | 0.004417453 | 1828.860316 |
| 81 | rs9516996 | G | C | 412181 | 0.0231353 | 0.104707 | finngen_R10_CIRRHOSIS_BROAD | 0.30518 | 6.01E-06 | 0.004649538 | 1925.394148 |
| 82 | rs1572592 | G | C | 412181 | 0.414723 | 1.87456 | finngen_R10_CIRRHOSIS_BROAD | 0.000230457 | 6.18E-06 | 0.001619267 | 668.5103929 |
| 83 | rs4982667 | T | A | 412181 | 0.0265143 | -0.12741 | finngen_R10_CIRRHOSIS_BROAD | 0.224439 | 1.54E-06 | 0.005651605 | 2342.712796 |
| 84 | rs183139663 | A | T | 412181 | 0.0838607 | -0.34358 | finngen_R10_CIRRHOSIS_BROAD | 0.0207682 | 4.18E-05 | 0.004801508 | 1988.629367 |
| 85 | rs17701869 | A | G | 412181 | 0.0331654 | -0.13611 | finngen_R10_CIRRHOSIS_BROAD | 0.130785 | 4.06E-05 | 0.004211943 | 1743.417485 |
| 86 | rs142894304 | T | C | 412181 | 0.0852274 | -0.35255 | finngen_R10_CIRRHOSIS_BROAD | 0.0199218 | 3.52E-05 | 0.004853646 | 2010.328526 |
| 87 | rs943322 | T | C | 412181 | 0.08832 | 0.389917 | finngen_R10_CIRRHOSIS_BROAD | 0.98074 | 1.01E-05 | 0.005743604 | 2381.068972 |
| 88 | rs1833173 | A | T | 412181 | 0.0266282 | 0.109199 | finngen_R10_CIRRHOSIS_BROAD | 0.779663 | 4.12E-05 | 0.00409696 | 1695.627603 |
| 89 | rs62030561 | G | A | 412181 | 0.0883723 | -0.36548 | finngen_R10_CIRRHOSIS_BROAD | 0.0195985 | 3.54E-05 | 0.005133151 | 2126.693629 |
| 90 | rs2589009 | T | C | 412181 | 0.0239468 | -0.0976 | finngen_R10_CIRRHOSIS_BROAD | 0.723611 | 4.58E-05 | 0.003810549 | 1576.636245 |
| 91 | rs117446369 | T | A | 412181 | 0.100058 | -0.45739 | finngen_R10_CIRRHOSIS_BROAD | 0.0155922 | 4.85E-06 | 0.006422285 | 2664.241485 |
| 92 | rs112093611 | G | A | 412181 | 0.0526475 | 0.219209 | finngen_R10_CIRRHOSIS_BROAD | 0.0386505 | 3.13E-05 | 0.003570945 | 1477.143397 |
| 93 | rs543301 | C | T | 412181 | 0.0218751 | 0.103552 | finngen_R10_CIRRHOSIS_BROAD | 0.555514 | 2.20E-06 | 0.005295416 | 2194.278839 |
| 94 | rs117667228 | A | G | 412181 | 0.107265 | 0.448767 | finngen_R10_CIRRHOSIS_BROAD | 0.00777868 | 2.87E-05 | 0.003108753 | 1285.358761 |
| 95 | rs1124736 | A | C | 412181 | 0.0229139 | -0.09543 | finngen_R10_CIRRHOSIS_BROAD | 0.348459 | 3.12E-05 | 0.004135126 | 1711.489249 |
| 96 | rs28431791 | T | C | 412181 | 0.02312 | 0.095309 | finngen_R10_CIRRHOSIS_BROAD | 0.306384 | 3.75E-05 | 0.003860867 | 1597.536127 |
| 97 | rs1393722 | C | T | 412181 | 0.0215889 | -0.08988 | finngen_R10_CIRRHOSIS_BROAD | 0.55619 | 3.14E-05 | 0.003988497 | 1650.557835 |
| 98 | rs4807713 | A | G | 412181 | 0.0333428 | -0.14723 | finngen_R10_CIRRHOSIS_BROAD | 0.129728 | 1.01E-05 | 0.004894667 | 2027.402417 |
| 99 | rs188247550 | T | C | 412181 | 0.0436871 | 0.407579 | finngen_R10_CIRRHOSIS_BROAD | 0.0508301 | 1.06E-20 | 0.016029446 | 6714.63292 |
| 100 | rs533229174 | A | C | 412181 | 0.0627562 | 0.278886 | finngen_R10_CIRRHOSIS_BROAD | 0.0257615 | 8.83E-06 | 0.00390409 | 1615.49107 |
| 101 | rs62238491 | T | C | 412181 | 0.0715652 | -0.31985 | finngen_R10_CIRRHOSIS_BROAD | 0.0286649 | 7.84E-06 | 0.005697019 | 2361.645842 |
| 102 | rs117574693 | T | C | 412181 | 0.14395 | -0.58569 | finngen_R10_CIRRHOSIS_BROAD | 0.00828344 | 4.73E-05 | 0.005635985 | 2336.201529 |
| 103 | rs5764023 | T | C | 412181 | 0.0252225 | 0.131828 | finngen_R10_CIRRHOSIS_BROAD | 0.225264 | 1.73E-07 | 0.006065839 | 2515.469724 |
| 104 | rs3747207 | A | G | 412181 | 0.0237257 | 0.393654 | finngen_R10_CIRRHOSIS_BROAD | 0.22649 | 7.98E-62 | 0.054296808 | 23664.93434 |
| 105 | rs4823188 | C | T | 412181 | 0.0359644 | 0.185268 | finngen_R10_CIRRHOSIS_BROAD | 0.0907461 | 2.59E-07 | 0.00566427 | 2347.992904 |

SNP, single nucleotide polymorphism; EA, effect allele; OA, other allele; EAF, effect allele frequency; SE, standard error.

**Supplementary Table S1.3** Characteristics of instrumental variables for liver cirrhosis and liver fibrosis

|  | **SNP** | **EA** | **OA** | **Samplesize** | **SE** | **β** | **id.exposure** | **EAF** | ***p* value** | **R^2^** | **F - statistic** |
| --- | --- | --- | --- | --- | --- | --- | --- | --- | --- | --- | --- |
| 1 | rs36004072 | A | G | 368291 | 0.120928 | 0.496169 | finngen_R9_K11_FIBROCHIRLIV | 0.0137147 | 4.08E-05 | 0.00666006 | 2469.272223 |
| 2 | rs279024 | A | G | 368291 | 0.0340517 | 0.142887 | finngen_R9_K11_FIBROCHIRLIV | 0.610761 | 2.71E-05 | 0.009707403 | 3610.175318 |
| 3 | rs1256328 | T | C | 368291 | 0.0564711 | -0.230391 | finngen_R9_K11_FIBROCHIRLIV | 0.108135 | 4.51E-05 | 0.010238266 | 3809.645012 |
| 4 | rs624698 | G | A | 368291 | 0.0347742 | 0.143378 | finngen_R9_K11_FIBROCHIRLIV | 0.320773 | 3.74E-05 | 0.008957932 | 3328.928266 |
| 5 | rs75560190 | C | A | 368291 | 0.0411026 | 0.166943 | finngen_R9_K11_FIBROCHIRLIV | 0.184519 | 4.87E-05 | 0.008387284 | 3115.071521 |
| 6 | rs114729724 | T | C | 368291 | 0.0903286 | 0.37346 | finngen_R9_K11_FIBROCHIRLIV | 0.027798 | 3.56E-05 | 0.007538557 | 2797.456528 |
| 7 | rs2051517 | G | C | 368291 | 0.0330276 | -0.138258 | finngen_R9_K11_FIBROCHIRLIV | 0.49217 | 2.84E-05 | 0.009555293 | 3553.059985 |
| 8 | rs13417424 | T | C | 368291 | 0.0961475 | -0.436239 | finngen_R9_K11_FIBROCHIRLIV | 0.0392359 | 5.70E-06 | 0.014347603 | 5360.981704 |
| 9 | rs414255 | T | C | 368291 | 0.0593018 | -0.24175 | finngen_R9_K11_FIBROCHIRLIV | 0.0979219 | 4.57E-05 | 0.010324926 | 3842.227247 |
| 10 | rs2218659 | A | G | 368291 | 0.0361796 | -0.155565 | finngen_R9_K11_FIBROCHIRLIV | 0.713682 | 1.71E-05 | 0.009890248 | 3678.854291 |
| 11 | rs7374418 | G | C | 368291 | 0.0514426 | -0.221058 | finngen_R9_K11_FIBROCHIRLIV | 0.130375 | 1.73E-05 | 0.011080741 | 4126.64129 |
| 12 | rs73126787 | A | G | 368291 | 0.0446482 | 0.189142 | finngen_R9_K11_FIBROCHIRLIV | 0.145385 | 2.27E-05 | 0.008889883 | 3303.413201 |
| 13 | rs6800736 | A | G | 368291 | 0.0333837 | 0.146862 | finngen_R9_K11_FIBROCHIRLIV | 0.385987 | 1.09E-05 | 0.010223489 | 3804.089451 |
| 14 | rs62286828 | T | C | 368291 | 0.0824646 | -0.337115 | finngen_R9_K11_FIBROCHIRLIV | 0.0523539 | 4.35E-05 | 0.011276683 | 4200.44536 |
| 15 | rs11726166 | G | A | 368291 | 0.0332318 | 0.14159 | finngen_R9_K11_FIBROCHIRLIV | 0.550337 | 2.04E-05 | 0.00992227 | 3690.884716 |
| 16 | rs114835516 | A | G | 368291 | 0.102514 | 0.42304 | finngen_R9_K11_FIBROCHIRLIV | 0.0204905 | 3.68E-05 | 0.007183797 | 2664.857327 |
| 17 | rs28636836 | T | C | 368291 | 0.0413138 | -0.201866 | finngen_R9_K11_FIBROCHIRLIV | 0.21707 | 1.03E-06 | 0.013850935 | 5172.795051 |
| 18 | rs28431971 | A | G | 368291 | 0.0430061 | -0.174657 | finngen_R9_K11_FIBROCHIRLIV | 0.192791 | 4.88E-05 | 0.009494558 | 3530.259424 |
| 19 | rs74616779 | T | G | 368291 | 0.098625 | -0.447864 | finngen_R9_K11_FIBROCHIRLIV | 0.0379185 | 5.60E-06 | 0.01463475 | 5469.867708 |
| 20 | rs77716303 | T | C | 368291 | 0.0589576 | 0.247186 | finngen_R9_K11_FIBROCHIRLIV | 0.0749616 | 2.76E-05 | 0.008473764 | 3147.464798 |
| 21 | rs511664 | G | A | 368291 | 0.039393 | -0.171618 | finngen_R9_K11_FIBROCHIRLIV | 0.790572 | 1.32E-05 | 0.009752857 | 3627.245846 |
| 22 | rs180802890 | T | G | 368291 | 0.480925 | 1.95625 | finngen_R9_K11_FIBROCHIRLIV | 0.000416801 | 4.75E-05 | 0.003188794 | 1178.154486 |
| 23 | rs75676059 | G | T | 368291 | 0.103474 | 0.452213 | finngen_R9_K11_FIBROCHIRLIV | 0.0198172 | 1.24E-05 | 0.00794448 | 2949.295044 |
| 24 | rs62401310 | G | A | 368291 | 0.0641315 | -0.267491 | finngen_R9_K11_FIBROCHIRLIV | 0.0846387 | 3.03E-05 | 0.011086894 | 4128.958241 |
| 25 | rs10463907 | T | C | 368291 | 0.119787 | 0.530475 | finngen_R9_K11_FIBROCHIRLIV | 0.013768 | 9.49E-06 | 0.007642048 | 2836.15642 |
| 26 | rs77062257 | T | A | 368291 | 0.0685304 | -0.32894 | finngen_R9_K11_FIBROCHIRLIV | 0.074171 | 1.59E-06 | 0.014860324 | 5555.44975 |
| 27 | rs191329273 | C | T | 368291 | 0.0503884 | 0.377404 | finngen_R9_K11_FIBROCHIRLIV | 0.0966008 | 6.89E-14 | 0.024860131 | 9389.127846 |
| 28 | rs62407980 | T | C | 368291 | 0.068725 | 0.342673 | finngen_R9_K11_FIBROCHIRLIV | 0.0501499 | 6.16E-07 | 0.011187033 | 4166.673857 |
| 29 | rs12665578 | T | C | 368291 | 0.0396599 | 0.192489 | finngen_R9_K11_FIBROCHIRLIV | 0.197917 | 1.21E-06 | 0.011763708 | 4384.016581 |
| 30 | rs71564122 | C | T | 368291 | 0.246163 | -1.01043 | finngen_R9_K11_FIBROCHIRLIV | 0.00842545 | 4.05E-05 | 0.017059289 | 6391.787999 |
| 31 | rs139598882 | A | G | 368291 | 0.607181 | 2.55333 | finngen_R9_K11_FIBROCHIRLIV | 0.000155722 | 2.61E-05 | 0.002030141 | 749.1996277 |
| 32 | rs117407510 | T | G | 368291 | 0.114556 | 0.501902 | finngen_R9_K11_FIBROCHIRLIV | 0.0159053 | 1.18E-05 | 0.007885815 | 2927.343557 |
| 33 | rs4876275 | T | G | 368291 | 0.033317 | -0.135237 | finngen_R9_K11_FIBROCHIRLIV | 0.482344 | 4.93E-05 | 0.00913312 | 3394.63137 |
| 34 | rs62484602 | A | G | 368291 | 0.0698711 | 0.291451 | finngen_R9_K11_FIBROCHIRLIV | 0.0491264 | 3.03E-05 | 0.007935948 | 2946.102549 |
| 35 | rs11993582 | G | T | 368291 | 0.0339105 | 0.147258 | finngen_R9_K11_FIBROCHIRLIV | 0.354197 | 1.41E-05 | 0.009920481 | 3690.212696 |
| 36 | rs11774268 | G | A | 368291 | 0.0489548 | -0.206728 | finngen_R9_K11_FIBROCHIRLIV | 0.146054 | 2.41E-05 | 0.010660375 | 3968.403368 |
| 37 | rs72659160 | T | C | 368291 | 0.503051 | 2.04853 | finngen_R9_K11_FIBROCHIRLIV | 0.000514289 | 4.66E-05 | 0.004314182 | 1595.750187 |
| 38 | rs13271471 | G | A | 368291 | 0.0362652 | 0.162133 | finngen_R9_K11_FIBROCHIRLIV | 0.689426 | 7.79E-06 | 0.011257076 | 4193.058729 |
| 39 | rs186592445 | T | G | 368291 | 0.182772 | 0.807389 | finngen_R9_K11_FIBROCHIRLIV | 0.00504893 | 9.99E-06 | 0.006549328 | 2427.94679 |
| 40 | rs2012276 | A | G | 368291 | 0.0388874 | -0.169363 | finngen_R9_K11_FIBROCHIRLIV | 0.783348 | 1.33E-05 | 0.009736089 | 3620.94817 |
| 41 | rs10814133 | A | G | 368291 | 0.0353452 | -0.177976 | finngen_R9_K11_FIBROCHIRLIV | 0.348333 | 4.77E-07 | 0.014380475 | 5373.44339 |
| 42 | rs112188193 | C | G | 368291 | 0.0910107 | 0.374565 | finngen_R9_K11_FIBROCHIRLIV | 0.0270883 | 3.86E-05 | 0.007395024 | 2743.796256 |
| 43 | rs176694 | G | T | 368291 | 0.0483771 | 0.208077 | finngen_R9_K11_FIBROCHIRLIV | 0.117632 | 1.70E-05 | 0.0089878 | 3340.128139 |
| 44 | rs116947616 | A | G | 368291 | 0.0863087 | 0.351949 | finngen_R9_K11_FIBROCHIRLIV | 0.0321976 | 4.55E-05 | 0.007719686 | 2865.194011 |
| 45 | rs11239729 | C | T | 368291 | 0.112052 | 0.483011 | finngen_R9_K11_FIBROCHIRLIV | 0.0163092 | 1.63E-05 | 0.00748575 | 2777.712565 |
| 46 | rs10902821 | A | G | 368291 | 0.0337661 | -0.13801 | finngen_R9_K11_FIBROCHIRLIV | 0.43091 | 4.37E-05 | 0.009341543 | 3472.829251 |
| 47 | rs145072712 | A | G | 368291 | 0.118295 | 0.509344 | finngen_R9_K11_FIBROCHIRLIV | 0.0146537 | 1.66E-05 | 0.007491841 | 2779.989997 |
| 48 | rs116859664 | T | C | 368291 | 0.247883 | -1.20365 | finngen_R9_K11_FIBROCHIRLIV | 0.00948783 | 1.20E-06 | 0.027230596 | 10309.46175 |
| 49 | rs191507130 | T | C | 368291 | 0.124948 | 0.507266 | finngen_R9_K11_FIBROCHIRLIV | 0.0131214 | 4.91E-05 | 0.00666416 | 2470.802619 |
| 50 | rs10879803 | T | A | 368291 | 0.0449243 | -0.18282 | finngen_R9_K11_FIBROCHIRLIV | 0.174642 | 4.71E-05 | 0.009635372 | 3583.126105 |
| 51 | rs2158241 | A | G | 368291 | 0.0349909 | -0.173418 | finngen_R9_K11_FIBROCHIRLIV | 0.354191 | 7.19E-07 | 0.013758147 | 5137.659004 |
| 52 | rs2475526 | G | A | 368291 | 0.0394907 | -0.183827 | finngen_R9_K11_FIBROCHIRLIV | 0.799269 | 3.24E-06 | 0.010843164 | 4037.19385 |
| 53 | rs74691952 | T | C | 368291 | 0.114214 | 0.476111 | finngen_R9_K11_FIBROCHIRLIV | 0.0154468 | 3.06E-05 | 0.006894839 | 2556.922984 |
| 54 | rs116891600 | A | G | 368291 | 0.171542 | -0.728079 | finngen_R9_K11_FIBROCHIRLIV | 0.014636 | 2.19E-05 | 0.015289951 | 5718.557277 |
| 55 | rs140503338 | G | T | 368291 | 0.0754479 | 0.331612 | finngen_R9_K11_FIBROCHIRLIV | 0.0406666 | 1.11E-05 | 0.00858021 | 3187.344927 |
| 56 | rs1289239 | A | G | 368291 | 0.0336383 | -0.165656 | finngen_R9_K11_FIBROCHIRLIV | 0.419188 | 8.45E-07 | 0.013362532 | 4987.924862 |
| 57 | rs12894872 | T | C | 368291 | 0.0501826 | 0.205173 | finngen_R9_K11_FIBROCHIRLIV | 0.109913 | 4.34E-05 | 0.008236675 | 3058.67009 |
| 58 | rs78347867 | A | T | 368291 | 0.143594 | 0.598664 | finngen_R9_K11_FIBROCHIRLIV | 0.00916031 | 3.06E-05 | 0.006505937 | 2411.755731 |
| 59 | rs146753693 | T | C | 368291 | 0.0959584 | -0.428996 | finngen_R9_K11_FIBROCHIRLIV | 0.0397323 | 7.80E-06 | 0.014043408 | 5245.700132 |
| 60 | rs767453 | T | A | 368291 | 0.0333582 | 0.151137 | finngen_R9_K11_FIBROCHIRLIV | 0.39721 | 5.88E-06 | 0.010938501 | 4073.082944 |
| 61 | rs77284671 | A | C | 368291 | 0.0786641 | 0.328074 | finngen_R9_K11_FIBROCHIRLIV | 0.0379777 | 3.04E-05 | 0.007864795 | 2919.478687 |
| 62 | rs45481191 | T | C | 368291 | 0.0581136 | 0.251254 | finngen_R9_K11_FIBROCHIRLIV | 0.075845 | 1.54E-05 | 0.008849683 | 3288.341864 |
| 63 | rs4787994 | A | G | 368291 | 0.0366441 | 0.150344 | finngen_R9_K11_FIBROCHIRLIV | 0.262028 | 4.08E-05 | 0.008741577 | 3247.81767 |
| 64 | rs75833872 | T | A | 368291 | 0.0549424 | -0.245387 | finngen_R9_K11_FIBROCHIRLIV | 0.114106 | 7.96E-06 | 0.012173721 | 4538.700357 |
| 65 | rs10083773 | G | C | 368291 | 0.0350281 | -0.14694 | finngen_R9_K11_FIBROCHIRLIV | 0.694081 | 2.73E-05 | 0.009169099 | 3408.127856 |
| 66 | rs17244536 | A | T | 368291 | 0.0531684 | 0.219168 | finngen_R9_K11_FIBROCHIRLIV | 0.0951803 | 3.75E-05 | 0.008273579 | 3072.488496 |
| 67 | rs117425352 | A | G | 368291 | 0.130556 | 0.609947 | finngen_R9_K11_FIBROCHIRLIV | 0.0108254 | 2.98E-06 | 0.007967666 | 2957.971759 |
| 68 | rs117865572 | A | G | 368291 | 0.0654559 | 0.288467 | finngen_R9_K11_FIBROCHIRLIV | 0.0576387 | 1.05E-05 | 0.009039697 | 3359.590787 |
| 69 | rs117456554 | T | C | 368291 | 0.106607 | 0.440577 | finngen_R9_K11_FIBROCHIRLIV | 0.0188108 | 3.58E-05 | 0.007165288 | 2657.941748 |
| 70 | rs4795843 | C | T | 368291 | 0.037773 | -0.156333 | finngen_R9_K11_FIBROCHIRLIV | 0.273869 | 3.49E-05 | 0.009720513 | 3615.098527 |
| 71 | rs8073734 | G | T | 368291 | 0.0439179 | 0.190226 | finngen_R9_K11_FIBROCHIRLIV | 0.152028 | 1.48E-05 | 0.009329854 | 3468.442615 |
| 72 | rs17072158 | C | G | 368291 | 0.0413286 | 0.179944 | finngen_R9_K11_FIBROCHIRLIV | 0.177848 | 1.34E-05 | 0.009469038 | 3520.679825 |
| 73 | rs247787 | A | G | 368291 | 0.039026 | -0.175365 | finngen_R9_K11_FIBROCHIRLIV | 0.789571 | 7.00E-06 | 0.010219099 | 3802.43933 |
| 74 | rs4813657 | G | C | 368291 | 0.0378099 | 0.167107 | finngen_R9_K11_FIBROCHIRLIV | 0.724285 | 9.89E-06 | 0.011152935 | 4153.830632 |
| 75 | rs62193453 | A | G | 368291 | 1.34521 | 6.13128 | finngen_R9_K11_FIBROCHIRLIV | 0.000159965 | 5.17E-06 | 0.012025075 | 4482.606466 |
| 76 | rs146454149 | A | G | 368291 | 0.100493 | 0.416725 | finngen_R9_K11_FIBROCHIRLIV | 0.021367 | 3.37E-05 | 0.007262606 | 2694.305758 |
| 77 | rs3747207 | A | G | 368291 | 0.0359682 | 0.421739 | finngen_R9_K11_FIBROCHIRLIV | 0.225534 | 9.45E-32 | 0.06213437 | 24399.4496 |
| 78 | rs150609497 | T | G | 368291 | 0.0645863 | -0.293689 | finngen_R9_K11_FIBROCHIRLIV | 0.0857095 | 5.44E-06 | 0.013518189 | 5046.824367 |

SNP, single nucleotide polymorphism; EA, effect allele; OA, other allele; EAF, effect allele frequency; SE, standard error.

**Supplementary Table S2.1** SNPs from GWAS on exposure (CHB) and outcome (T2D)

|  |  | | | | **Exposure (CHB)** | | |  | | **Outcome (T2D)** | | | | |
| --- | --- | --- | --- | --- | --- | --- | --- | --- | --- | --- | --- | --- | --- | --- |
|  | **SNP** | **EA** | **OA** | **β** | | **SE** | ***p* value** |  | **Case** | | **Control** | **β** | **SE** | ***p* value** |
| 1 | rs10283724 | T | C | -0.27063 | | 0.0664273 | 4.62E-05 |  | 77,418 | | 356,122 | 0.0169 | 0.0136 | 0.214000112 |
| 2 | rs1038744 | A | G | -0.207233 | | 0.0496923 | 3.04E-05 |  | 77,418 | | 356,122 | 0.0162 | 0.0087 | 0.062589286 |
| 3 | rs10483245 | T | A | -0.24512 | | 0.0559042 | 1.16E-05 |  | 77,418 | | 356,122 | -0.0106 | 0.0099 | 0.284300092 |
| 4 | rs10814940 | G | C | 0.303951 | | 0.0646476 | 2.58E-06 |  | 77,418 | | 356,122 | -0.0117 | 0.0104 | 0.260599753 |
| 5 | rs115183117 | C | G | -0.76187 | | 0.0994431 | 1.84E-14 |  | 77,418 | | 356,122 | 0.0571 | 0.0201 | 0.004499974 |
| 6 | rs11889341 | T | C | -0.213492 | | 0.0413158 | 2.37E-07 |  | 77,418 | | 356,122 | -0.0058 | 0.0072 | 0.4205 |
| 7 | rs12184995 | G | A | -0.307495 | | 0.0745566 | 3.72E-05 |  | 77,418 | | 356,122 | -0.0037 | 0.0143 | 0.795799936 |
| 8 | rs12417665 | T | C | -0.301759 | | 0.0727445 | 3.35E-05 |  | 77,418 | | 356,122 | 0.0138 | 0.0134 | 0.303100044 |
| 9 | rs12530346 | G | A | 0.466509 | | 0.105879 | 1.05E-05 |  | 77,418 | | 356,122 | 0.014 | 0.0175 | 0.423699547 |
| 10 | rs12813856 | T | C | 0.190644 | | 0.044127 | 1.56E-05 |  | 77,418 | | 356,122 | -0.0102 | 0.0075 | 0.173800091 |
| 11 | rs12919083 | C | A | 0.220315 | | 0.0451168 | 1.04E-06 |  | 77,418 | | 356,122 | 0.002 | 0.0076 | 0.792399148 |
| 12 | rs13118152 | A | G | 1.75228 | | 0.414197 | 2.33E-05 |  | 77,418 | | 356,122 | -0.0163 | 0.0404 | 0.686600318 |
| 13 | rs1473639 | T | C | 0.165926 | | 0.0398703 | 3.16E-05 |  | 77,418 | | 356,122 | -0.015 | 0.0076 | 0.048419467 |
| 14 | rs1477495 | A | C | 0.216089 | | 0.0505638 | 1.92E-05 |  | 77,418 | | 356,122 | 0.0097 | 0.0086 | 0.259400017 |
| 15 | rs148472310 | T | G | 0.800492 | | 0.180346 | 9.05E-06 |  | 77,418 | | 356,122 | 0.0523 | 0.0275 | 0.057189987 |
| 16 | rs149893379 | A | C | -0.621793 | | 0.105466 | 3.73E-09 |  | 77,418 | | 356,122 | 0.0083 | 0.0221 | 0.70719959 |
| 17 | rs16953371 | T | C | 0.258305 | | 0.0528411 | 1.02E-06 |  | 77,418 | | 356,122 | -0.0096 | 0.0097 | 0.322299773 |
| 18 | rs1718789 | G | T | 0.456981 | | 0.0898452 | 3.65E-07 |  | 77,418 | | 356,122 | -0.0141 | 0.0155 | 0.363000313 |
| 19 | rs201177682 | C | A | 0.171378 | | 0.0407488 | 2.60E-05 |  | 77,418 | | 356,122 | -0.0063 | 0.0071 | 0.37489962 |
| 20 | rs2074043 | A | G | 0.166053 | | 0.0386206 | 1.71E-05 |  | 77,418 | | 356,122 | -0.0025 | 0.0067 | 0.708999616 |
| 21 | rs2302969 | A | G | -0.262897 | | 0.057438 | 4.72E-06 |  | 77,418 | | 356,122 | 0.0209 | 0.0109 | 0.055179784 |
| 22 | rs2788381 | A | C | -0.344524 | | 0.0788673 | 1.25E-05 |  | 77,418 | | 356,122 | -0.0092 | 0.0136 | 0.498699577 |
| 23 | rs2925999 | C | T | 0.183066 | | 0.0434623 | 2.53E-05 |  | 77,418 | | 356,122 | -0.004 | 0.008 | 0.617100645 |
| 24 | rs34996023 | A | G | 1.00498 | | 0.231217 | 1.38E-05 |  | 77,418 | | 356,122 | -0.0115 | 0.0476 | 0.809099998 |
| 25 | rs3821426 | G | C | 0.220563 | | 0.0506994 | 1.36E-05 |  | 77,418 | | 356,122 | 0.0041 | 0.0084 | 0.625499548 |
| 26 | rs4623766 | A | T | 0.388497 | | 0.0917267 | 2.28E-05 |  | 77,418 | | 356,122 | -0.0053 | 0.0154 | 0.730699819 |
| 27 | rs4660036 | A | G | 0.170285 | | 0.0382738 | 8.62E-06 |  | 77,418 | | 356,122 | -0.0157 | 0.0068 | 0.020949806 |
| 28 | rs6062784 | A | G | 0.209591 | | 0.0497503 | 2.52E-05 |  | 77,418 | | 356,122 | -0.009 | 0.0094 | 0.338300002 |
| 29 | rs67753410 | T | C | 0.388416 | | 0.0950659 | 4.39E-05 |  | 77,418 | | 356,122 | -0.0047 | 0.0157 | 0.764699841 |
| 30 | rs6914573 | T | A | 0.579461 | | 0.0505987 | 2.30E-30 |  | 77,418 | | 356,122 | -0.0299 | 0.0091 | 0.001016998 |
| 31 | rs6997303 | T | C | 0.255547 | | 0.0620755 | 3.84E-05 |  | 77,418 | | 356,122 | 0.0128 | 0.0106 | 0.227199829 |
| 32 | rs7011514 | C | A | 0.182822 | | 0.0447543 | 4.41E-05 |  | 77,418 | | 356,122 | -0.0055 | 0.0082 | 0.502400427 |
| 33 | rs73081274 | C | A | 0.863862 | | 0.197656 | 1.24E-05 |  | 77,418 | | 356,122 | -0.0066 | 0.0409 | 0.871800071 |
| 34 | rs75455415 | T | C | 0.650728 | | 0.153755 | 2.31E-05 |  | 77,418 | | 356,122 | -0.0339 | 0.0222 | 0.126799926 |
| 35 | rs78192747 | T | A | -0.573659 | | 0.11913 | 1.47E-06 |  | 77,418 | | 356,122 | 0.0725 | 0.0271 | 0.007467066 |

SNP, single nucleotide polymorphism; EA, effect allele; OA, other allele; SE, standard error; CHB, chronic hepatitis B; T2D, type 2 diabetes.

**Supplementary Table S2.2** SNPs from GWAS on exposure (liver cirrhosis) and outcome (T2D)

|  |  | | | | **Exposure (liver cirrhosis)** | | |  | | **Outcome (T2D)** | | | | |
| --- | --- | --- | --- | --- | --- | --- | --- | --- | --- | --- | --- | --- | --- | --- |
|  | **SNP** | **EA** | **OA** | **β** | | **SE** | ***p* value** |  | **Case** | | **Control** | **β** | **SE** | ***p* value** |
| 1 | rs10803185 | T | C | 0.120244 | | 0.0296457 | 4.99E-05 |  | 57,698 | | 308,252 | 0.0194952 | 0.00963659 | 0.0430695 |
| 2 | rs11062525 | T | C | 0.1264 | | 0.0293752 | 1.69E-05 |  | 57,698 | | 308,252 | -0.016476 | 0.00953098 | 0.0838668 |
| 3 | rs11118610 | C | A | 0.0978294 | | 0.0215647 | 5.72E-06 |  | 57,698 | | 308,252 | 0.00530751 | 0.00683785 | 0.437633 |
| 4 | rs111227872 | A | G | 0.50509 | | 0.11988 | 2.52E-05 |  | 57,698 | | 308,252 | -0.00283872 | 0.0452101 | 0.949934 |
| 5 | rs112093611 | G | A | 0.219209 | | 0.0526475 | 3.13E-05 |  | 57,698 | | 308,252 | 0.00818822 | 0.0179012 | 0.647375 |
| 6 | rs1124736 | A | C | -0.0954295 | | 0.0229139 | 3.12E-05 |  | 57,698 | | 308,252 | -0.0158561 | 0.00720401 | 0.0277351 |
| 7 | rs112786023 | T | C | -0.321839 | | 0.0783249 | 3.97E-05 |  | 57,698 | | 308,252 | 0.00886959 | 0.0223314 | 0.691234 |
| 8 | rs114297714 | T | C | 0.270902 | | 0.0580439 | 3.05E-06 |  | 57,698 | | 308,252 | 0.0164342 | 0.0194416 | 0.397939 |
| 9 | rs114628928 | G | A | -0.416221 | | 0.0904261 | 4.17E-06 |  | 57,698 | | 308,252 | 0.0442891 | 0.025134 | 0.0780495 |
| 10 | rs115203506 | A | G | -0.233948 | | 0.0500996 | 3.02E-06 |  | 57,698 | | 308,252 | -0.0100274 | 0.0149088 | 0.501214 |
| 11 | rs115248419 | G | A | 0.281388 | | 0.0593561 | 2.13E-06 |  | 57,698 | | 308,252 | 0.0062996 | 0.0203894 | 0.757349 |
| 12 | rs115346713 | A | G | 0.252934 | | 0.0607036 | 3.09E-05 |  | 57,698 | | 308,252 | 0.0612451 | 0.0204028 | 0.00268392 |
| 13 | rs116073620 | C | T | 0.542646 | | 0.127068 | 1.95E-05 |  | 57,698 | | 308,252 | 0.0141461 | 0.0470914 | 0.763874 |
| 14 | rs117574693 | T | C | -0.585694 | | 0.14395 | 4.73E-05 |  | 57,698 | | 308,252 | -0.00798886 | 0.0381148 | 0.83398 |
| 15 | rs117725374 | G | C | -0.577486 | | 0.141748 | 4.62E-05 |  | 57,698 | | 308,252 | -0.0440103 | 0.0379704 | 0.246429 |
| 16 | rs118007202 | C | T | 0.324092 | | 0.07444 | 1.34E-05 |  | 57,698 | | 308,252 | 0.0174732 | 0.0260862 | 0.50297 |
| 17 | rs12407185 | T | C | -0.325985 | | 0.0785094 | 3.29E-05 |  | 57,698 | | 308,252 | 0.00315499 | 0.0223602 | 0.887792 |
| 18 | rs12515587 | G | T | -0.159522 | | 0.0356271 | 7.55E-06 |  | 57,698 | | 308,252 | -0.0159227 | 0.0108208 | 0.141157 |
| 19 | rs13253177 | T | G | 0.127324 | | 0.0307817 | 3.53E-05 |  | 57,698 | | 308,252 | 0.00646381 | 0.0100366 | 0.51956 |
| 20 | rs138242499 | T | C | 0.307225 | | 0.0622397 | 7.97E-07 |  | 57,698 | | 308,252 | 0.0109583 | 0.0214559 | 0.609537 |
| 21 | rs139113551 | T | C | -0.576078 | | 0.134299 | 1.79E-05 |  | 57,698 | | 308,252 | -0.0545197 | 0.0352925 | 0.122396 |
| 22 | rs139347990 | T | C | 0.502305 | | 0.105776 | 2.05E-06 |  | 57,698 | | 308,252 | 0.0258808 | 0.0387683 | 0.504403 |
| 23 | rs1393722 | C | T | -0.0898834 | | 0.0215889 | 3.14E-05 |  | 57,698 | | 308,252 | -0.0158085 | 0.00686227 | 0.0212408 |
| 24 | rs140274847 | T | C | 0.298939 | | 0.0725626 | 3.79E-05 |  | 57,698 | | 308,252 | -0.00807512 | 0.0252606 | 0.749218 |
| 25 | rs140529475 | T | A | -0.504351 | | 0.121708 | 3.41E-05 |  | 57,698 | | 308,252 | -0.0128747 | 0.0328213 | 0.694861 |
| 26 | rs141733595 | G | A | -0.937585 | | 0.216437 | 1.48E-05 |  | 57,698 | | 308,252 | 0.0662331 | 0.0517191 | 0.200324 |
| 27 | rs141862829 | A | G | -2.30384 | | 0.53116 | 1.44E-05 |  | 57,698 | | 308,252 | -0.121574 | 0.0880985 | 0.167595 |
| 28 | rs142633515 | T | G | 0.272993 | | 0.0560548 | 1.12E-06 |  | 57,698 | | 308,252 | -0.00345191 | 0.0190993 | 0.856575 |
| 29 | rs142706535 | C | A | -0.283084 | | 0.0639196 | 9.48E-06 |  | 57,698 | | 308,252 | 0.000458019 | 0.0187091 | 0.980469 |
| 30 | rs142838457 | A | C | -1.18018 | | 0.289682 | 4.62E-05 |  | 57,698 | | 308,252 | -0.152032 | 0.0619885 | 0.0141837 |
| 31 | rs142894304 | T | C | -0.352553 | | 0.0852274 | 3.52E-05 |  | 57,698 | | 308,252 | 0.00832728 | 0.0244938 | 0.733875 |
| 32 | rs143555556 | G | C | 0.193489 | | 0.0463523 | 2.99E-05 |  | 57,698 | | 308,252 | -0.0116357 | 0.0157718 | 0.460664 |
| 33 | rs147275274 | A | G | 0.316295 | | 0.0656159 | 1.43E-06 |  | 57,698 | | 308,252 | -0.0085274 | 0.0229906 | 0.710706 |
| 34 | rs147602787 | G | A | -0.5075 | | 0.123035 | 3.71E-05 |  | 57,698 | | 308,252 | -0.0545015 | 0.0332784 | 0.101475 |
| 35 | rs147763991 | G | A | -0.425742 | | 0.0887322 | 1.60E-06 |  | 57,698 | | 308,252 | -0.00966695 | 0.0247203 | 0.695758 |
| 36 | rs147773046 | T | C | -1.19233 | | 0.283089 | 2.53E-05 |  | 57,698 | | 308,252 | 0.0728116 | 0.0607149 | 0.230436 |
| 37 | rs149331187 | T | C | -0.525066 | | 0.125253 | 2.76E-05 |  | 57,698 | | 308,252 | -0.0635893 | 0.0339037 | 0.0607128 |
| 38 | rs1700406 | A | T | -0.231874 | | 0.0540629 | 1.80E-05 |  | 57,698 | | 308,252 | -0.00853739 | 0.0160772 | 0.595403 |
| 39 | rs17701869 | A | G | -0.136108 | | 0.0331654 | 4.06E-05 |  | 57,698 | | 308,252 | 0.0056408 | 0.0101386 | 0.577959 |
| 40 | rs182826156 | A | C | -0.496034 | | 0.120143 | 3.65E-05 |  | 57,698 | | 308,252 | 0.0278369 | 0.032696 | 0.394555 |
| 41 | rs183139663 | A | T | -0.343583 | | 0.0838607 | 4.18E-05 |  | 57,698 | | 308,252 | 0.00472492 | 0.0240134 | 0.844014 |
| 42 | rs186749123 | C | T | 0.315216 | | 0.0771106 | 4.35E-05 |  | 57,698 | | 308,252 | 0.0138444 | 0.0265261 | 0.601726 |
| 43 | rs192057253 | A | C | 0.394037 | | 0.0935595 | 2.54E-05 |  | 57,698 | | 308,252 | -0.022515 | 0.0325671 | 0.48935 |
| 44 | rs2589009 | T | C | -0.0976036 | | 0.0239468 | 4.58E-05 |  | 57,698 | | 308,252 | 0.0113493 | 0.00769789 | 0.14039 |
| 45 | rs2841538 | A | G | 0.291609 | | 0.0715776 | 4.62E-05 |  | 57,698 | | 308,252 | 0.018796 | 0.0244737 | 0.442481 |
| 46 | rs28431791 | T | C | 0.0953092 | | 0.02312 | 3.75E-05 |  | 57,698 | | 308,252 | -0.00637228 | 0.00741207 | 0.389945 |
| 47 | rs28488125 | T | C | 0.125997 | | 0.0297561 | 2.29E-05 |  | 57,698 | | 308,252 | 0.00677679 | 0.00969876 | 0.484722 |
| 48 | rs28636836 | T | C | -0.222029 | | 0.0271778 | 3.10E-16 |  | 57,698 | | 308,252 | 0.0114618 | 0.0082517 | 0.164826 |
| 49 | rs2869726 | G | A | -0.124598 | | 0.0228758 | 5.13E-08 |  | 57,698 | | 308,252 | 0.0133552 | 0.00735263 | 0.0693123 |
| 50 | rs2926217 | C | T | 0.0919821 | | 0.0215059 | 1.89E-05 |  | 57,698 | | 308,252 | -0.00127713 | 0.00681912 | 0.851437 |
| 51 | rs34118288 | A | T | -0.100385 | | 0.0239236 | 2.72E-05 |  | 57,698 | | 308,252 | -0.00559688 | 0.00767901 | 0.466091 |
| 52 | rs35065486 | A | G | -0.112267 | | 0.0245022 | 4.61E-06 |  | 57,698 | | 308,252 | -0.00380119 | 0.00762255 | 0.618008 |
| 53 | rs35930927 | T | C | 0.103859 | | 0.0248473 | 2.92E-05 |  | 57,698 | | 308,252 | 0.0196607 | 0.00798521 | 0.0138108 |
| 54 | rs3747207 | A | G | 0.393654 | | 0.0237257 | 7.98E-62 |  | 57,698 | | 308,252 | 0.0169579 | 0.00814859 | 0.0374257 |
| 55 | rs4728409 | C | T | 0.102165 | | 0.0235203 | 1.40E-05 |  | 57,698 | | 308,252 | -0.0046547 | 0.0075542 | 0.53778 |
| 56 | rs4762535 | G | C | -0.0960963 | | 0.0220369 | 1.30E-05 |  | 57,698 | | 308,252 | 0.00416116 | 0.00692682 | 0.548019 |
| 57 | rs4807713 | A | G | -0.147232 | | 0.0333428 | 1.01E-05 |  | 57,698 | | 308,252 | 0.000486471 | 0.0101488 | 0.961769 |
| 58 | rs4823188 | C | T | 0.185268 | | 0.0359644 | 2.59E-07 |  | 57,698 | | 308,252 | -0.0132203 | 0.0119438 | 0.26835 |
| 59 | rs4976286 | A | C | -0.132648 | | 0.0306841 | 1.54E-05 |  | 57,698 | | 308,252 | 0.00380696 | 0.00942647 | 0.686316 |
| 60 | rs4982667 | T | A | -0.127413 | | 0.0265143 | 1.54E-06 |  | 57,698 | | 308,252 | 0.000343994 | 0.00818567 | 0.96648 |
| 61 | rs533229174 | A | C | 0.278886 | | 0.0627562 | 8.83E-06 |  | 57,698 | | 308,252 | 0.00149997 | 0.021667 | 0.944808 |
| 62 | rs543301 | C | T | 0.103552 | | 0.0218751 | 2.20E-06 |  | 57,698 | | 308,252 | 0.00490442 | 0.00692246 | 0.478648 |
| 63 | rs56058442 | T | C | 0.111396 | | 0.0230186 | 1.30E-06 |  | 57,698 | | 308,252 | -0.002946 | 0.00737638 | 0.689611 |
| 64 | rs5764023 | T | C | 0.131828 | | 0.0252225 | 1.73E-07 |  | 57,698 | | 308,252 | 0.00141869 | 0.00819679 | 0.862589 |
| 65 | rs59664098 | A | G | 0.196382 | | 0.0441348 | 8.60E-06 |  | 57,698 | | 308,252 | 0.00868068 | 0.0147334 | 0.555738 |
| 66 | rs61634079 | G | A | 0.0918948 | | 0.022194 | 3.47E-05 |  | 57,698 | | 308,252 | -0.00555204 | 0.00699995 | 0.427688 |
| 67 | rs61946210 | T | C | -0.134776 | | 0.0326558 | 3.67E-05 |  | 57,698 | | 308,252 | -0.00342323 | 0.0100371 | 0.733059 |
| 68 | rs62030561 | G | A | -0.36548 | | 0.0883723 | 3.54E-05 |  | 57,698 | | 308,252 | -0.061242 | 0.0245985 | 0.0127865 |
| 69 | rs62238491 | T | C | -0.319852 | | 0.0715652 | 7.84E-06 |  | 57,698 | | 308,252 | 0.010085 | 0.0205384 | 0.623403 |
| 70 | rs6774537 | T | C | -0.0916821 | | 0.0215797 | 2.15E-05 |  | 57,698 | | 308,252 | -0.0103192 | 0.00683867 | 0.131313 |
| 71 | rs6815416 | T | C | -0.122138 | | 0.0298633 | 4.32E-05 |  | 57,698 | | 308,252 | -0.0102236 | 0.00920276 | 0.266599 |
| 72 | rs6858066 | G | A | 0.108233 | | 0.0217149 | 6.22E-07 |  | 57,698 | | 308,252 | 0.00599509 | 0.00686661 | 0.38262 |
| 73 | rs6994761 | G | A | 0.0965092 | | 0.0217344 | 8.98E-06 |  | 57,698 | | 308,252 | 0.0110121 | 0.00690575 | 0.110794 |
| 74 | rs72758827 | A | T | 0.175432 | | 0.0408777 | 1.77E-05 |  | 57,698 | | 308,252 | 0.0226128 | 0.0134423 | 0.0925273 |
| 75 | rs72796530 | T | C | 0.178628 | | 0.042136 | 2.24E-05 |  | 57,698 | | 308,252 | 0.00888341 | 0.0139854 | 0.525303 |
| 76 | rs73212521 | G | C | -0.228501 | | 0.0551774 | 3.45E-05 |  | 57,698 | | 308,252 | 0.01401 | 0.0163143 | 0.390474 |
| 77 | rs73241837 | A | T | -0.106132 | | 0.0219895 | 1.39E-06 |  | 57,698 | | 308,252 | 0.0028852 | 0.00691639 | 0.676566 |
| 78 | rs74440280 | A | C | 0.554716 | | 0.132966 | 3.02E-05 |  | 57,698 | | 308,252 | 0.00936473 | 0.051412 | 0.855465 |
| 79 | rs74590923 | A | G | -0.790963 | | 0.184783 | 1.86E-05 |  | 57,698 | | 308,252 | -0.0694487 | 0.0457352 | 0.128889 |
| 80 | rs74620573 | A | C | 0.734516 | | 0.157809 | 3.25E-06 |  | 57,698 | | 308,252 | -0.0197756 | 0.0626753 | 0.752362 |
| 81 | rs75323042 | C | T | -0.389804 | | 0.085486 | 5.12E-06 |  | 57,698 | | 308,252 | 0.0291577 | 0.0240668 | 0.225691 |
| 82 | rs75407439 | T | A | 0.374057 | | 0.0872245 | 1.80E-05 |  | 57,698 | | 308,252 | 0.00905466 | 0.0312795 | 0.772216 |
| 83 | rs76954838 | T | C | 1.37282 | | 0.320979 | 1.89E-05 |  | 57,698 | | 308,252 | -0.0870601 | 0.158304 | 0.58235 |
| 84 | rs77838486 | T | A | 0.324516 | | 0.0780984 | 3.25E-05 |  | 57,698 | | 308,252 | -0.00455074 | 0.0269404 | 0.865861 |
| 85 | rs77990616 | G | A | 1.46343 | | 0.346933 | 2.46E-05 |  | 57,698 | | 308,252 | 0.206372 | 0.1742 | 0.236142 |
| 86 | rs7803171 | A | G | -0.115067 | | 0.0270287 | 2.07E-05 |  | 57,698 | | 308,252 | -0.0140786 | 0.00837831 | 0.0928859 |
| 87 | rs78071553 | T | C | 0.545978 | | 0.120119 | 5.49E-06 |  | 57,698 | | 308,252 | 0.0755557 | 0.0445444 | 0.0898504 |
| 88 | rs78091895 | G | A | -0.306446 | | 0.0730063 | 2.70E-05 |  | 57,698 | | 308,252 | -0.000147682 | 0.0210957 | 0.994414 |
| 89 | rs78349710 | C | T | -0.197144 | | 0.0413737 | 1.89E-06 |  | 57,698 | | 308,252 | -0.00391951 | 0.0124099 | 0.752125 |
| 90 | rs79020788 | T | C | -0.289575 | | 0.0690246 | 2.73E-05 |  | 57,698 | | 308,252 | 0.0356204 | 0.0199861 | 0.0747068 |
| 91 | rs80196313 | C | T | 0.400915 | | 0.0914436 | 1.16E-05 |  | 57,698 | | 308,252 | -0.00916198 | 0.0323242 | 0.776839 |
| 92 | rs846906 | C | T | 0.123307 | | 0.0297206 | 3.34E-05 |  | 57,698 | | 308,252 | 0.0057146 | 0.00913258 | 0.531487 |
| 93 | rs943322 | T | C | 0.389917 | | 0.08832 | 1.01E-05 |  | 57,698 | | 308,252 | 0.0227601 | 0.0250164 | 0.362923 |
| 94 | rs9516996 | G | C | 0.104707 | | 0.0231353 | 6.02E-06 |  | 57,698 | | 308,252 | -0.00455425 | 0.00743036 | 0.539926 |

SNP, single nucleotide polymorphism; EA, effect allele; OA, other allele; SE, standard error; T2D, type 2 diabetes.

**Supplementary Table S2.3** SNPs from GWAS on exposure (liver cirrhosis and liver fibrosis) and outcome (T2D)

|  |  | | | | **Exposure (liver cirrhosis and liver fibrosis)** | | |  | | **Outcome (T2D)** | | | | |
| --- | --- | --- | --- | --- | --- | --- | --- | --- | --- | --- | --- | --- | --- | --- |
|  | **SNP** | **EA** | **OA** | **β** | | **SE** | ***p* value** |  | **Case** | | **Control** | **β** | **SE** | ***p* value** |
| 1 | rs10083773 | G | C | -0.14694 | | 0.0350281 | 2.73E-05 |  | 57,698 | | 308,252 | -0.012424 | 0.00740098 | 0.0932116 |
| 2 | rs10463907 | T | C | 0.530475 | | 0.119787 | 9.49E-06 |  | 57,698 | | 308,252 | -0.0110593 | 0.0293742 | 0.706549 |
| 3 | rs10814133 | A | G | -0.177976 | | 0.0353452 | 4.77E-07 |  | 57,698 | | 308,252 | -0.0154498 | 0.00720669 | 0.0320487 |
| 4 | rs10879803 | T | A | -0.18282 | | 0.0449243 | 4.71E-05 |  | 57,698 | | 308,252 | 0.00183029 | 0.00895202 | 0.837998 |
| 5 | rs10902821 | A | G | -0.13801 | | 0.0337661 | 4.37E-05 |  | 57,698 | | 308,252 | 0.00152345 | 0.0069485 | 0.826457 |
| 6 | rs112188193 | C | G | 0.374565 | | 0.0910107 | 3.86E-05 |  | 57,698 | | 308,252 | -0.0241017 | 0.0212268 | 0.256193 |
| 7 | rs11239729 | C | T | 0.483011 | | 0.112052 | 1.63E-05 |  | 57,698 | | 308,252 | 0.0482088 | 0.0273103 | 0.0775247 |
| 8 | rs114729724 | T | C | 0.37346 | | 0.0903286 | 3.56E-05 |  | 57,698 | | 308,252 | 0.0255137 | 0.0209112 | 0.222428 |
| 9 | rs114835516 | A | G | 0.42304 | | 0.102514 | 3.68E-05 |  | 57,698 | | 308,252 | -0.02192 | 0.0241613 | 0.364281 |
| 10 | rs116859664 | T | C | -1.20365 | | 0.247883 | 1.20E-06 |  | 57,698 | | 308,252 | 0.061132 | 0.0362897 | 0.0920746 |
| 11 | rs116891600 | A | G | -0.728079 | | 0.171542 | 2.19E-05 |  | 57,698 | | 308,252 | 0.0267342 | 0.0288028 | 0.353313 |
| 12 | rs116947616 | A | G | 0.351949 | | 0.0863087 | 4.55E-05 |  | 57,698 | | 308,252 | -0.00219976 | 0.0198784 | 0.911885 |
| 13 | rs11726166 | G | A | 0.14159 | | 0.0332318 | 2.04E-05 |  | 57,698 | | 308,252 | -0.00579733 | 0.00686239 | 0.398225 |
| 14 | rs117407510 | T | G | 0.501902 | | 0.114556 | 1.18E-05 |  | 57,698 | | 308,252 | -0.0327548 | 0.0279579 | 0.241367 |
| 15 | rs117425352 | A | G | 0.609947 | | 0.130556 | 2.98E-06 |  | 57,698 | | 308,252 | 0.00628045 | 0.0331915 | 0.849922 |
| 16 | rs117456554 | T | C | 0.440577 | | 0.106607 | 3.58E-05 |  | 57,698 | | 308,252 | -0.00628948 | 0.0253407 | 0.803982 |
| 17 | rs11774268 | G | A | -0.206728 | | 0.0489548 | 2.41E-05 |  | 57,698 | | 308,252 | 0.0144908 | 0.00968175 | 0.134468 |
| 18 | rs117865572 | A | G | 0.288467 | | 0.0654559 | 1.05E-05 |  | 57,698 | | 308,252 | 0.0228062 | 0.0146798 | 0.120286 |
| 19 | rs11993582 | G | T | 0.147258 | | 0.0339105 | 1.41E-05 |  | 57,698 | | 308,252 | 0.00757074 | 0.00713575 | 0.288709 |
| 20 | rs1256328 | T | C | -0.230391 | | 0.0564711 | 4.51E-05 |  | 57,698 | | 308,252 | -0.0163361 | 0.0110265 | 0.138465 |
| 21 | rs12665578 | T | C | 0.192489 | | 0.0396599 | 1.21E-06 |  | 57,698 | | 308,252 | 0.000201973 | 0.00853016 | 0.98111 |
| 22 | rs1289239 | A | G | -0.165656 | | 0.0336383 | 8.45E-07 |  | 57,698 | | 308,252 | -0.00177289 | 0.00692549 | 0.797955 |
| 23 | rs12894872 | T | C | 0.205173 | | 0.0501826 | 4.34E-05 |  | 57,698 | | 308,252 | -0.0108698 | 0.0109696 | 0.321733 |
| 24 | rs13271471 | G | A | 0.162133 | | 0.0362652 | 7.79E-06 |  | 57,698 | | 308,252 | 0.00701078 | 0.00736932 | 0.341429 |
| 25 | rs13417424 | T | C | -0.436239 | | 0.0961475 | 5.70E-06 |  | 57,698 | | 308,252 | 0.00527821 | 0.0175588 | 0.763718 |
| 26 | rs139598882 | A | G | 2.55333 | | 0.607181 | 2.61E-05 |  | 57,698 | | 308,252 | 0.472299 | 0.282782 | 0.0948833 |
| 27 | rs145072712 | A | G | 0.509344 | | 0.118295 | 1.66E-05 |  | 57,698 | | 308,252 | 0.0143431 | 0.0287237 | 0.617534 |
| 28 | rs146753693 | T | C | -0.428996 | | 0.0959584 | 7.80E-06 |  | 57,698 | | 308,252 | -0.0274082 | 0.0175464 | 0.118277 |
| 29 | rs150609497 | T | G | -0.293689 | | 0.0645863 | 5.44E-06 |  | 57,698 | | 308,252 | 0.0131644 | 0.0123961 | 0.288245 |
| 30 | rs17072158 | C | G | 0.179944 | | 0.0413286 | 1.34E-05 |  | 57,698 | | 308,252 | -0.00625558 | 0.00890407 | 0.482334 |
| 31 | rs17244536 | A | T | 0.219168 | | 0.0531684 | 3.75E-05 |  | 57,698 | | 308,252 | 0.0176654 | 0.0116503 | 0.129442 |
| 32 | rs186592445 | T | G | 0.807389 | | 0.182772 | 9.99E-06 |  | 57,698 | | 308,252 | -0.076968 | 0.0494718 | 0.119757 |
| 33 | rs191329273 | C | T | 0.377404 | | 0.0503884 | 6.89E-14 |  | 57,698 | | 308,252 | -0.00372421 | 0.0115831 | 0.747815 |
| 34 | rs191507130 | T | C | 0.507266 | | 0.124948 | 4.91E-05 |  | 57,698 | | 308,252 | 0.0353577 | 0.0300823 | 0.239848 |
| 35 | rs2012276 | A | G | -0.169363 | | 0.0388874 | 1.33E-05 |  | 57,698 | | 308,252 | -0.00971731 | 0.00830074 | 0.241737 |
| 36 | rs2218659 | A | G | -0.155565 | | 0.0361796 | 1.71E-05 |  | 57,698 | | 308,252 | -0.00700905 | 0.00767123 | 0.360885 |
| 37 | rs2475526 | G | A | -0.183827 | | 0.0394907 | 3.24E-06 |  | 57,698 | | 308,252 | -0.00615472 | 0.00849381 | 0.46869 |
| 38 | rs279024 | A | G | 0.142887 | | 0.0340517 | 2.71E-05 |  | 57,698 | | 308,252 | -0.00854533 | 0.00698292 | 0.221048 |
| 39 | rs28431971 | A | G | -0.174657 | | 0.0430061 | 4.88E-05 |  | 57,698 | | 308,252 | -0.0121779 | 0.0086353 | 0.158467 |
| 40 | rs28636836 | T | C | -0.201866 | | 0.0413138 | 1.03E-06 |  | 57,698 | | 308,252 | 0.0114618 | 0.0082517 | 0.164826 |
| 41 | rs36004072 | A | G | 0.496169 | | 0.120928 | 4.08E-05 |  | 57,698 | | 308,252 | -0.0341222 | 0.0294852 | 0.247164 |
| 42 | rs3747207 | A | G | 0.421739 | | 0.0359682 | 9.45E-32 |  | 57,698 | | 308,252 | 0.0169579 | 0.00814859 | 0.0374257 |
| 43 | rs414255 | T | C | -0.24175 | | 0.0593018 | 4.57E-05 |  | 57,698 | | 308,252 | -0.0125913 | 0.011565 | 0.27627 |
| 44 | rs45481191 | T | C | 0.251254 | | 0.0581136 | 1.54E-05 |  | 57,698 | | 308,252 | 0.0234496 | 0.0129356 | 0.0698634 |
| 45 | rs4787994 | A | G | 0.150344 | | 0.0366441 | 4.08E-05 |  | 57,698 | | 308,252 | 0.00222452 | 0.00778003 | 0.774935 |
| 46 | rs4795843 | C | T | -0.156333 | | 0.037773 | 3.49E-05 |  | 57,698 | | 308,252 | -0.00977306 | 0.00764441 | 0.201087 |
| 47 | rs4813657 | G | C | 0.167107 | | 0.0378099 | 9.89E-06 |  | 57,698 | | 308,252 | -0.00769379 | 0.00766553 | 0.31553 |
| 48 | rs4876275 | T | G | -0.135237 | | 0.033317 | 4.93E-05 |  | 57,698 | | 308,252 | -0.0101021 | 0.00689651 | 0.142971 |
| 49 | rs511664 | G | A | -0.171618 | | 0.039393 | 1.32E-05 |  | 57,698 | | 308,252 | -0.012666 | 0.00844532 | 0.133676 |
| 50 | rs62401310 | G | A | -0.267491 | | 0.0641315 | 3.03E-05 |  | 57,698 | | 308,252 | -0.0103249 | 0.0123981 | 0.404966 |
| 51 | rs62407980 | T | C | 0.342673 | | 0.068725 | 6.16E-07 |  | 57,698 | | 308,252 | 0.0216141 | 0.0158116 | 0.171633 |
| 52 | rs624698 | G | A | 0.143378 | | 0.0347742 | 3.74E-05 |  | 57,698 | | 308,252 | 0.000598147 | 0.00731719 | 0.934849 |
| 53 | rs62484602 | A | G | 0.291451 | | 0.0698711 | 3.03E-05 |  | 57,698 | | 308,252 | 0.00414853 | 0.0157575 | 0.792339 |
| 54 | rs6800736 | A | G | 0.146862 | | 0.0333837 | 1.09E-05 |  | 57,698 | | 308,252 | 0.00834781 | 0.00699065 | 0.232423 |
| 55 | rs71564122 | C | T | -1.01043 | | 0.246163 | 4.05E-05 |  | 57,698 | | 308,252 | 0.00956785 | 0.0378639 | 0.800507 |
| 56 | rs73126787 | A | G | 0.189142 | | 0.0446482 | 2.27E-05 |  | 57,698 | | 308,252 | -0.00202653 | 0.00966465 | 0.833914 |
| 57 | rs74616779 | T | G | -0.447864 | | 0.098625 | 5.60E-06 |  | 57,698 | | 308,252 | 0.0109078 | 0.0178924 | 0.542104 |
| 58 | rs74691952 | T | C | 0.476111 | | 0.114214 | 3.06E-05 |  | 57,698 | | 308,252 | -0.0278779 | 0.0276826 | 0.313909 |
| 59 | rs75560190 | C | A | 0.166943 | | 0.0411026 | 4.87E-05 |  | 57,698 | | 308,252 | 0.00888547 | 0.00879066 | 0.31212 |
| 60 | rs75676059 | G | T | 0.452213 | | 0.103474 | 1.24E-05 |  | 57,698 | | 308,252 | 0.0306861 | 0.0246717 | 0.213582 |
| 61 | rs75833872 | T | A | -0.245387 | | 0.0549424 | 7.96E-06 |  | 57,698 | | 308,252 | -0.0105218 | 0.0107277 | 0.326689 |
| 62 | rs767453 | T | A | 0.151137 | | 0.0333582 | 5.88E-06 |  | 57,698 | | 308,252 | -0.011156 | 0.00698595 | 0.110284 |
| 63 | rs77062257 | T | A | -0.32894 | | 0.0685304 | 1.59E-06 |  | 57,698 | | 308,252 | -0.0139204 | 0.013001 | 0.284294 |
| 64 | rs77284671 | A | C | 0.328074 | | 0.0786641 | 3.04E-05 |  | 57,698 | | 308,252 | 0.0289375 | 0.0179842 | 0.107606 |
| 65 | rs77716303 | T | C | 0.247186 | | 0.0589576 | 2.76E-05 |  | 57,698 | | 308,252 | -0.0108405 | 0.0131125 | 0.408389 |
| 66 | rs78347867 | A | T | 0.598664 | | 0.143594 | 3.06E-05 |  | 57,698 | | 308,252 | 0.0529153 | 0.0364599 | 0.146689 |
| 67 | rs8073734 | G | T | 0.190226 | | 0.0439179 | 1.48E-05 |  | 57,698 | | 308,252 | 0.0156547 | 0.00951018 | 0.0997447 |

SNP, single nucleotide polymorphism; EA, effect allele; OA, other allele; SE, standard error; T2D, type 2 diabetes.

**Supplementary Table S3** The results of MR-Egger intercept analysis

| **Exposure** | **Outcome** | **Race** | **Egger_intercept** | **SE** | ***p* value** |
| --- | --- | --- | --- | --- | --- |
| CHB | T2D | East Asian | 0.000177139 | 0.004376381 | 0.967957395 |
| Liver cirrhosis | T2D | European | -0.000164142 | 0.002342496 | 0.94428901 |
| Liver cirrhosis and liver fibrosis | T2D | European | 0.003823723 | 0.003261677 | 0.245348264 |

*CHB, chronic hepatitis B; T2D, type 2 diabetes.

**Supplementary Table S4** The results of heterogeneity analysis

| **Exposure** | **Outcome** | **Race** | **Method** | **Q** | **Q_df** | **Q_*p* val** |
| --- | --- | --- | --- | --- | --- | --- |
| CHB | T2D | East Asian | MR Egger | 48.07712715 | 33 | 0.043617908 |
| CHB | T2D | East Asian | Inverse variance weighted | 48.07951399 | 34 | 0.055398803 |
| Liver cirrhosis | T2D | European | MR Egger | 108.1366084 | 92 | 0.120017925 |
| Liver cirrhosis | T2D | European | Inverse variance weighted | 108.1423796 | 93 | 0.134914445 |
| Liver cirrhosis and liver fibrosis | T2D | European | MR Egger | 81.34426027 | 65 | 0.082912086 |
| Liver cirrhosis and liver fibrosis | T2D | European | Inverse variance weighted | 83.06416459 | 66 | 0.076279044 |

*CHB, chronic hepatitis B; T2D, type 2 diabetes.

**Supplementary Table S5** Literature report of included SNPs.

| **SNP** | **Report** | **Source** |
| --- | --- | --- |
| rs10483245 | The rs10483245 is related to the significant increase of vertical cup-to-disk ratio in cases of primary open angle glaucoma in South India. | DOI: 10.1002/mgg3.290 |
| rs1256328 | The study subjects with minor TT genotype at rs1256328 (alkaline phosphatase, liver/bone/kidney (ALPL)) have higher susceptibility to nephrolithiasis (OR 2.03, *p* = 0.0013). | [DOI: 10.1038/s41598-019-53261-8](https://doi.org/10.1038/s41598-019-53261-8) |
| rs11889341 | STAT1-STAT4 locus is the first genetic locus related to lupus risk and one of the highest multiple loci. In B cell lines of lupus patients and healthy controls, the lupus risk allele of rs11889341 is associated with the increased expression of STAT1. | [DOI: 10.1093/hmg/ddy140](https://doi.org/10.1093/hmg/ddy140) |
| rs846906 | Compared with reference genotype, carriers of the rs846906-T allele had significantly higher waist circumference and triglycerides and lower high-density lipoprotein-cholesterol exclusively in men (*p* = 0.028). The rs846906-T allele was also associated with a higher risk of MetS at 3 months of follow-up (OR 3.31, 95% CI 1.53–7.17). | [DOI: 10.1097/FPC.0000000000000131](https://doi.org/10.1097/FPC.0000000000000131) |
| rs6858066 | The highly significant interaction between LPHN3 tag SNPs(rs 6858066) and maternal pressure during pregnancy. | [DOI: 10.1111/j.1469-7610.2012.02551.x](https://doi.org/10.1111/j.1469-7610.2012.02551.x) |
| rs3747207 | Subjects aged more than 55 years with rs3747207 GA were more likely to develop hepatocellular carcinoma. | [DOI: 10.1186/s12920-022-01394-7](https://doi.org/10.1186/s12920-022-01394-7) |

* SNPs, single nucleotide polymorphisms.
